# High-Cost Cancer Drug Use in Medicare Advantage and Traditional Medicare

**DOI:** 10.1001/jamahealthforum.2024.4868

**Published:** 2025-01-10

**Authors:** Cathy J. Bradley, Rifei Liang, Richard C. Lindrooth, Lindsay M. Sabik, Marcelo C. Perraillon

**Affiliations:** 1 Department of Health Systems, Management, and Policy, University of Colorado Cancer Center, Aurora; 2 Department of Health Systems, Management, and Policy, Colorado School of Public Health, Aurora; 3 Department of Health Policy and Management, University of Pittsburgh School of Public Health, Pittsburgh, Pennsylvania

## Abstract

**Importance:**

Medicare Advantage (MA) plans are designed to incentivize the use of less expensive drugs through capitated payments, formulary control, and preauthorizations for certain drugs. These conditions may reduce spending on high-cost therapies for conditions such as cancer, a condition that is among the most expensive to treat.

**Objective:**

To determine whether patients insured by MA plans receive less high-cost drugs than those insured by traditional Medicare (TM).

**Design, Setting, and Participants:**

This cohort study used data from the linked Colorado All Payer Claims Database and Colorado Central Cancer Registry. This population-based cohort included adults 65 years and older insured by Medicare with prescription coverage who reside in Colorado and were diagnosed with colorectal (CRC) or non–small cell lung cancer (NSCLC) between January 2012 and December 2021. The data were analyzed between December 2023 and August 2024.

**Exposure:**

Enrollment in TM or MA insurance plans.

**Main Outcomes and Measures:**

Claims for chemotherapy and oral targeted agents were identified. Thresholds for high-cost drugs were based on the distribution of drug costs. Inverse probability weighted logistic regression for receiving any cancer drug and for receiving a high-cost cancer drug was estimated, controlling for patient and ecological characteristics. The sample was stratified by cancer site and local/regional and distant stage.

**Results:**

Of 4240 patients included in the analysis (mean [SD] age, 75 [7] years; 2327 [54.9%] female), 1991 were diagnosed with CRC and 2249 with NSCLC. A total of 1647 patients had local or regional CRC, and 344 had distant CRC; 1351 patients had local or regional NSCLC, and 898 had distant NSCLC. In the covariate-adjusted analysis, patients diagnosed with local or regional CRC who were insured by MA were 6.0 percentage points less likely to receive a cancer drug than similar patients insured by TM. Patients diagnosed with distant NSCLC were 10.0 percentage points less likely to receive a cancer drug if insured by MA. Among patients who received a cancer drug, patients insured by MA were less likely to receive a high-cost drug for local or regional CRC (by 10.0 percentage points) and distant CRC (by 9.0 percentage points).

**Conclusions and Relevance:**

In this cohort study, high-cost drugs were more commonly prescribed among patients enrolled in TM and diagnosed with CRC. A similar pattern was not observed for patients with NSCLC, perhaps because clinical evidence suggests survival benefits to be associated only with certain drugs, all of which are expensive. Nonetheless, MA was modestly associated with reduced high-cost drug utilization and may reduce overall treatment costs.

## Key Points

**Question:**

Are patients enrolled in Medicare Advantage (MA), compared to those enrolled in traditional Medicare (TM), less likely to be prescribed a high-cost drug for cancer treatment?

**Findings:**

In this cohort study of 4240 patients with colorectal cancer (CRC) or non–small cell lung cancer (NSCLC), those with local or regional CRC who were insured by MA were less likely to receive a cancer drug, and of those patients, were less likely to receive a high-cost cancer drug than similar patients who were insured by TM. Patients diagnosed with distant NSCLC were less likely to receive a cancer drug if insured by MA compared to TM.

**Meaning:**

MA appears to reduce high-cost drug utilization to treat patients with CRC, but not to treat those with NSCLC, in which few low-cost treatments exist.

## Teaser

This cohort study evaluates whether patients insured by Medicare Advantage plans receive less high-cost drugs than those insured by traditional Medicare.

## Introduction

Traditional Medicare’s (TM) fee-for-service reimbursement encourages clinicians to provide higher-cost care, including prescribing expensive drugs when similar less expensive drugs are available.^1,2^ Medicare Advantage (MA) plans, where beneficiaries receive managed care almost exclusively from in-network hospitals and clinicians, were designed to reduce costs by paying a risk-adjusted capitated amount per member. MA plans also have drug formularies where they control drugs that can be prescribed; they often require step-therapy to treat some conditions and preauthorizations for some prescription drugs.^3^ MA plans are growing in popularity with approximately 54% of Medicare beneficiaries enrolled in MA plans in 2024.^4^ Evidence suggests that MA plans are associated with more preventive care^5,6^ and with lower cost prescribing patterns of Part B and Part D drugs for conditions like diabetes where similar, less expensive alternatives exist.^7,8^

Cancer drugs are among the most expensive drugs in the US, leading to considerable financial burden among patients and payers.^9^ Effective approaches to controlling treatment costs for patients with cancer are elusive. The Oncology Care Model attempted to reduce costs of care,^10^ but an evaluation of the program reported that participating practices fell short of anticipated goals, partly due to the failure to control drug costs, leading to recommendations to hold clinicians accountable for inappropriate drug utilization.^11^ First-line therapies for some cancers, for example, platinum-based chemotherapy for non–small cell lung cancer (NSCLC), are less expensive than second-line and third-line alternatives such as targeted tyrosine kinase inhibitors where costs can exceed $10 000 a month.^12^ These newer drugs may lead to prolonged progression-free survival compared to older therapies.^13^ Thus, there may not be similar or interchangeable alternatives. Given their incentives to control cost, and the available evidence in other diseases, MA plans may still dampen, using prior authorization or formularies, the tendency for clinicians to prescribe higher-cost drugs. The composite effect of MA incentives on oncology prescribing, given the enthusiasm for newer therapies, remains an empirical question. The consequences of these practices on patient survival are unclear.

Research to date has not examined whether medications differ between patients diagnosed with cancer for those enrolled in MA compared to TM. To address this question, we used Colorado All Payer Claims Database (APCD) linked with the Colorado Central Cancer Registry (CCCR) to provide evidence derived from Part B and Part D drugs, as well as supplemental prescription drug plans, to assess whether patients insured by MA are more likely to receive lower-cost drugs compared to patients insured by TM. This unique dataset has claims data from TM and MA plans in addition to claims from supplemental prescription drug plans, which are not available in claims released by the Centers for Medicare & Medicaid Services.

We consider 2 cancer sites, colorectal cancer (CRC) and NSCLC. We chose these sites because they are common cancers; and when diagnosed with distant disease, mortality rates are high. New, targeted, and expensive therapies have recently been approved for NSCLC. Targeted therapies such as osimertinib,^14^ lorlatinib,^15^ and selpercatinib^16^ became available in the past 10 years for NSCLC and are believed to be superior to lower-cost alternatives. In addition, these therapies are believed to be responsible for lower mortality among patients with NSCLC. Less expensive therapies remain effective for CRC.^17^ Our findings inform whether MA approaches to cost control can reduce utilization of high-cost drugs.

## Methods

### Data Sources

This retrospective cohort study was reviewed and deemed exempt by the Colorado Multiple Institutional Review Board. The requirement for written informed consent was waived because the data were deidentified. We use linked data from January 2012 to December 2021 from the Colorado APCD and CCCR, a dataset comprising adults 21 years and older diagnosed with cancer in the CCCR and matched to the APCD. The overall linkage rate was 93%. The linkage rate for adults insured by TM and MA was 98% and 99%, respectively. Prior published assessments of validity concluded that the linked data were of high quality,^18^ and APCD data on treatment were highly reliable. Comparisons of the number of TM claims and MA encounter claims found similar treatment rates for multiple cancer sites, adding confidence that the fee-for-service claims and MA encounter data are similarly complete, which agrees with other assessments of Medicare-registry linkages.^19,20^ The Strengthening the Reporting of Observational Studies in Epidemiology (STROBE) reporting guideline was followed.

### Cohort Selection

We identified patients 65 years and older diagnosed with a first and only primary CRC or NSCLC from January 2012 to December 2021. We excluded patients who were not linked to the APCD or diagnosed through an autopsy or death certificate and patients who were enrolled in a plan other than Medicare or did not have continuous enrollment 3 months after diagnosis. In addition, we required patients to have at least 1 medical claim and 1 pharmacy claim during the 12 months following diagnosis. Although patients were enrolled in either TM or MA, we included claims from all health plans to capture supplemental coverage that may reimburse prescription medications.

Few managed care enrollees with CRC or NSCLC resided in rural areas of the state, in part because fewer MA plans are offered in rural areas in Colorado^21^ and these areas are sparsely populated, leading to small sample sizes. Therefore, we excluded patients who did not live in metropolitan counties or had missing zip codes. Rural residents diagnosed with cancer are more likely to be diagnosed at a later stage,^22^ have greater risk factors such as smoking and obesity, and are more likely to be older and White relative to urban residents diagnosed with cancer.^23,24,25^ Therefore, by removing rural residents we also reduced the heterogeneity in the sample and inherent selection into the TM or MA.

In our assessment of treatments prescribed, we stratified by local or regional and distant disease and excluded patients diagnosed with in situ and unstaged, unknown, or unspecified stage cancer. This distinction is important because standard protocols call for less expensive first- and second-line therapies for treatment with local or regional intent of each cancer.^26^ Following the failure of these treatments (for example, fluorouracil/leucovorin), newer, more expensive options are recommended. Palliative care in lieu of active treatment is an option for patients although mostly offered to those with late-stage disease. We further excluded patients who switched plans (TM to MA or vice versa) during the study period; only 16 patients switched from MA to TM, and 29 patients switched from TM to MA.

We used the Surveillance, Epidemiology, and End Results (SEER) summary staging system because TNM (tumor, node, metastasis) data were only available for approximately 50% of the sample. For patients with SEER summary staging and TNM staging, there was good agreement (κ coefficient of 0.93) when combining TNM stage I, II, and III into local/regional and TNM stage IV into distant. By excluding patients with unknown or unstaged disease, we disproportionately reduced the distant stage sample because later-stage disease is more likely categorized as unstaged or unknown.

### Cancer-Directed Treatment and High-Cost Treatment

We investigated 2 main outcomes. First, we examined whether patients received any cancer-directed drug. Second, we examined whether the patient received a high-cost drug, conditional on receiving a cancer-directed drug. We included all claims for systemic anticancer agents, including oral and infusion therapies and immunotherapies. Following prior studies,^27,28^ the reference standard for measuring treatment is APCD claims because registry data only capture initial treatment. We set a threshold for high-cost CRC drugs as those exceeding $6500 per month (the 25th percentile) among patients who received treatment. Similarly, we set the threshold at $8000 for NSCLC (eTables 1 and 2 in Supplement 1). We conducted sensitivity analyses using a range of high-cost definitions that covered the 25th to the 75th percentile (eTables 7 and 8 in Supplement 1). The National Drug Codes and Healthcare Common Procedure Coding System codes used to define agents were obtained from the Cancer Medications Enquiry Database available in the SEER Observational Research in Oncology Toolbox (eTable 3 in Supplement 1).^29^ Drug costs were derived from the Drug Pricing Lab website.^30^ The Drug Pricing Lab cost reflects the regimen for an average adult weighing 70 kg, or with a body surface area of 1.7 m^2^. The drug-specific monthly cost estimate was compared against the thresholds to define a high-cost drug. This approach is more standardized and is independent of negotiated prices and rebates paid by clinicians, health systems, and insurers, and bundled payment models.

The follow-up period was any time after the month of diagnosis, allowing for recovery from surgery and/or failure of first-line or second-line therapies that may be less expensive and progression to more expensive targeted agents. To determine whether we had comparable observation periods for patients enrolled in MA and those enrolled in TM, we compared observation months across groups. We determined survival months by using vital status and the last contact date data extracted from the CCCR. In sensitivity analyses, we restricted the follow-up time to 12 months for all patients (eTables 4 and 5 in Supplement 1).

### Covariates

We controlled for sex (male or female) and race and ethnicity (non-Hispanic White, non-Hispanic Black, Hispanic, and other [Alaska Native; American Indian; Asian; Hispanic, unknown race; multiracial; or Pacific Islander]). The source for race and ethnicity information was CCCR data, which was abstracted from clinical records as reported by patients. We included these variables to control for differences in treatment patterns related to structural racism. We included variables for patient age (65-74 years and ≥75 years) at diagnosis. Using the 12 months prior to diagnosis, we incorporated categorical variables representing the number of comorbidities (0, 1, or ≥2), as determined by the 2021 Charlson Comorbidity Index^31^ that tracks the presence of 16 specific conditions, marital status (not married or partnered, married or partnered), and observation months. Within the local or regional samples, we included a variable for local stage or regional stage according to the SEER summary stage. We conducted a sensitivity analysis excluding local stage disease from the sample because these patients may not be candidates for anticancer drugs; the results were robust (eTable 6 in Supplement 1). We also included a variable for Medicaid enrollment that may indicate poor health status and/or low income. All models controlled for observation follow-up time.

We included several ecological variables identified by the Agency for Healthcare Research and Quality as social determinants of health that may partially control for differences between people who enrolled in an MA or TM plan.^32^ These variables included the percentage of the population who were 65 years and older, Gini index of income inequality (values range from 0 [perfect equality] to 1 [perfect inequality]), per capita inflation-adjusted income, and percentage of the population with less than a high school education at the zip code tabulation area. We also included each patient’s travel time calculating miles to the nearest medical surgical intensive care unit from the estimated zip code centroid. In addition, we included the percentage of MA plans at the county level and MA market penetration as these factors may be associated with plan selection.^33^

### Statistical Analysis

We compared descriptive characteristics between TM and MA by site and SEER summary stage using χ^2^ tests to determine statistical significance. Prior studies suggest that MA beneficiaries are healthier than beneficiaries enrolled in TM.^34,35^ To reduce potential bias when comparing the TM sample to the MA sample, we estimated inverse probability weighted regression.^36^ Inverse probability weighted regression uses a logistic regression model to estimate the probability of MA enrollment, conditional on covariates. The inverse of the predicted probability of MA enrollment (ie, the propensity score) was used as a weight in covariate-adjusted outcome models to further balance model covariates among patients enrolled in TM and MA. eFigures 1 and 2 in Supplement 1 show balance plots of standardized mean differences before and after weighting across TM and MA. For ease of interpretation, we reported marginal effects, which are interpreted as average differences in the probability of getting high-cost agents between TM and MA.^37^ In sensitivity analyses, we restricted the follow-up time to 12 months for both groups and changed the thresholds to define high-cost drugs based on the distribution of the drug price (eTables 4-9 in Supplement 1). We used the implementation of inverse probability weighted regression in Stata 18 statistical software (StataCorp LLC), which simultaneously estimated the 2-step models to calculate asymptotically valid standard errors. *P* values less than .05 were considered statistically significant with 2-sided testing. The data were analyzed between December 2023 and August 2024.

## Results

Of 4240 patients included in the analysis (mean [SD] age, 75 [7] years; 2327 [54.9%] female), 1991 were diagnosed with CRC, and 2249 were diagnosed with NSCLC. The Figure shows the number of patients excluded from the analysis by reason. Of those analyzed, 1647 patients had local or regional CRC, and 344 had distant CRC; 1351 patients had local or regional NSCLC, and 898 had distant NSCLC.

**Figure.**
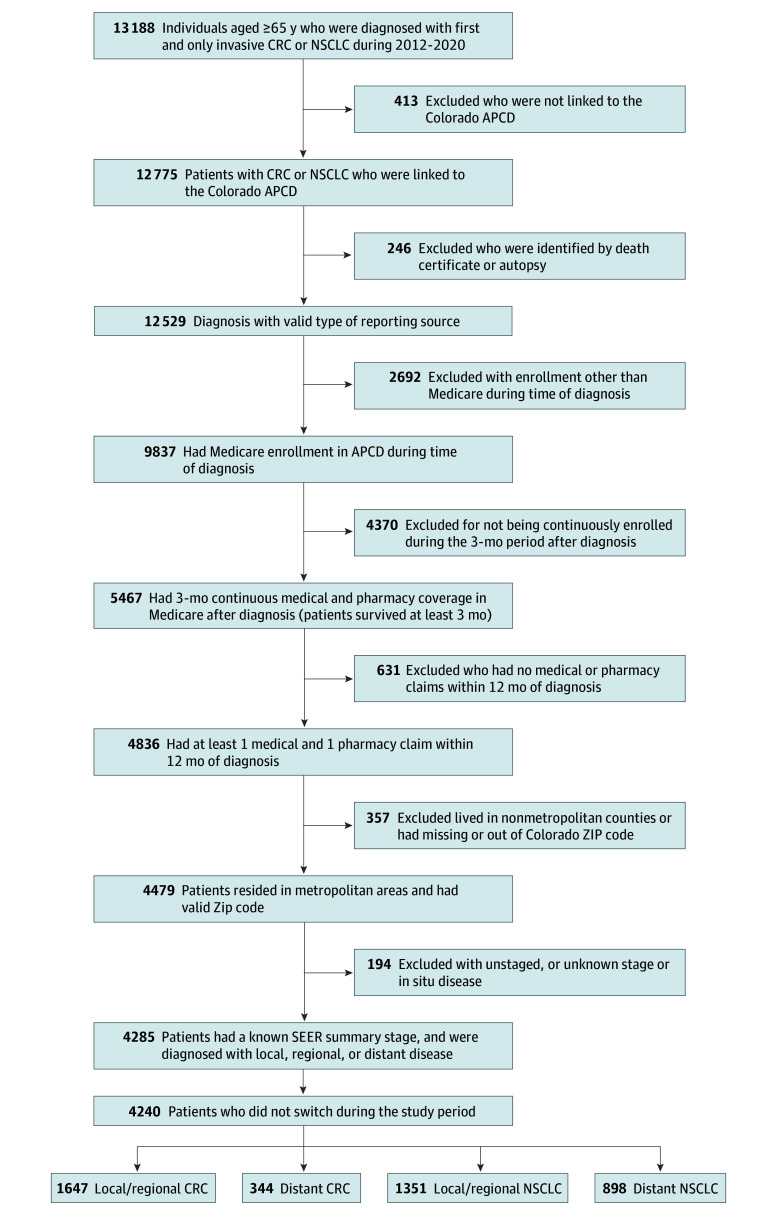
Study Flowchart for Patients With Colorectal Cancer (CRC) and Non–Small Cell Lung Cancer (NSCLC) Study data were obtained retrospectively from the Colorado All Payer Claims Database (APCD) and Colorado Central Cancer Registry. The data spanned from January 2012 to December 2021. The Surveillance, Epidemiology, and End Results (SEER) summary staging system was used to categorize people into local, regional, or distant disease.

Among patients diagnosed with local or regional CRC, those enrolled in MA had fewer comorbidities than patients enrolled in TM (Table 1).^38^ No statistically significant differences were observed between TM and MA enrollees diagnosed with distant CRC. Patients enrolled in TM lived in areas with a greater proportion of people 65 years and older and a lower MA penetration rate with a similar number of MA plans available. A higher proportion of patients in Medicaid were also enrolled in TM. Patients enrolled in MA were more likely to be Black and Hispanic.

**Table 1.** Characteristics of Patients With Colorectal and Non–Small Cell Lung Cancer by Stage and Medicare Plan

Characteristic	Colorectal cancer (n = 1991)						Non–small cell lung cancer (n = 2249)					
	Local/regional (n = 1647)			Distant (n = 344)			Local/regional (n = 1351)			Distant (n = 898)		
	TM (n = 542)	MA (n = 1105)	*P* value	TM (n = 135)	MA (n = 209)	*P* value	TM (n = 462)	MA (n = 889)	*P* value	TM (n = 306)	MA (n = 592)	*P* value
**No. (%)**												
Age group, y												
65-74	299 (55.2)	531 (48.1)	.01	80 (59.3)	107 (51.2)	.14	273 (59.1)	456 (51.3)	.01	188 (61.4)	303 (51.2)	.003
≥75	243 (44.8)	574 (51.9)		55 (40.7)	102 (48.8)		189 (40.9)	433 (48.7)		118 (38.6)	289 (48.8)	
Sex												
Female	302 (55.7)	578 (52.3)	.19	62 (45.9)	104 (49.8)	.49	256 (55.4)	527 (59.3)	.17	167 (54.6)	331 (55.9)	.70
Male	240 (44.3)	527 (47.7)		73 (54.1)	105 (50.2)		206 (44.6)	362 (40.7)		139 (45.4)	261 (44.1)	
Race and ethnicity												
White, non-Hispanic	469 (86.5)	899 (81.4)	.01	109 (80.7)	173 (82.8)	.63	418 (90.5)	773 (87.0)	.06	264 (86.3)	504 (85.1)	.65
Other^a^	73 (13.5)	206 (18.6)		26 (19.3)	36 (17.2)		44 (9.5)	116 (13.0)		42 (13.7)	88 (14.9)	
Comorbidities												
0	206 (38.0)	473 (42.8)	.01	52 (38.5)	84 (40.2)	.15	92 (19.9)	216 (24.3)	.06	115 (37.6)	231 (39.0)	.49
1	141 (26.0)	214 (19.4)		41 (30.4)	45 (21.5)		137 (29.7)	217 (24.4)		77 (25.2)	128 (21.6)	
≥2	195 (36.0)	418 (37.8)		42 (31.1)	80 (38.3)		233 (50.4)	456 (51.3)		114 (37.3)	233 (39.4)	
Marital status												
Not married or partnered	246 (45.4)	502 (45.4)	.99	65 (48.1)	106 (50.7)	.64	207 (44.8)	439 (49.4)	.11	132 (43.1)	280 (47.3)	.24
Married or partnered	296 (54.6)	603 (54.6)		70 (51.9)	103 (49.3)		255 (55.2)	450 (50.6)		174 (56.9)	312 (52.7)	
Medicare and Medicaid (dually eligible)												
No	473 (87.3)	1057 (95.7)	<.001	115 (85.2)	195 (93.3)	.01	401 (86.8)	816 (91.8)	.004	259 (84.6)	567 (95.8)	<.001
Yes	69 (12.7)	48 (4.3)		20 (14.8)	14 (6.7)		61 (13.2)	73 (8.2)		47 (15.4)	25 (4.2)	
**Mean (SD)**												
Observation months^b^	11.5 (1.7)	11.4 (1.9)	.56	9.8 (3.1)	9.6 (3.3)	.76	10.8 (2.6)	10.9 (2.5)	.42	8.9 (3.4)	8.7 (3.5)	.56
Survival months^c^	11.7 (1.3)	11.6 (1.6)	.78	9.9 (3.1)	9.7 (3.3)	.16	11.1 (2.3)	11.2 (2.2)	.39	9.2 (3.4)	8.9 (3.5)	.91
Population aged ≥65 y, %^d^	14.7 (5.3)	13.9 (4.8)	.001	15.2 (5.5)	13.9 (5.0)	.02	14.9 (5.7)	13.8 (4.7)	<.001	14.8 (4.9)	13.7 (4.9)	.003
Gini index^d^	0.4 (0.1)	0.4 (0.1)	.06	0.4 (0.1)	0.4 (0.1)	.60	0.4 (0.1)	0.4 (0.1)	.03	0.4 (0.1)	0.4 (0.1)	.03
Per capita income^d^	35 855 (12 566)	35 523 (11 578)	.61	36 298 (12 155)	35 542 (11 587)	.56	36 711 (12 425)	35 303 (10 952)	.04	36 239 (12 518)	36 031 (12 013)	.81
Population with <high school education, %^d^	8.3 (6.9)	9.2 (7.7)	.02	7.8 (6.4)	9.2 (7.7)	.07	7.8 (6.2)	9.2 (7.5)	<.001	8.3 (6.6)	9.0 (7.9)	.14
Miles to nearest medical-surgical ICU^d^	4.7 (5.4)	4.1 (4.6)	.01	5.3 (6.3)	4.5 (4.8)	.20	5.0 (5.9)	4.1 (4.8)	.01	5.1 (6.5)	4.4 (4.5)	.09
MA plans in county, %^e^	0.02 (0.002)	0.02 (0.001)	<.001	0.02 (0.002)	0.02 (0.001)	<.001	0.02 (0.002)	0.02 (0.001)	<.001	0.02 (0.002)	0.02 (0.001)	<.001
Penetration rate of MA plans in county	0.08 (0.05)	0.10 (0.05)	<.001	0.08 (0.05)	0.10 (0.05)	<.001	0.08 (0.05)	0.10 (0.05)	<.001	0.08 (0.05)	0.10 (0.05)	<.001

Abbreviations: ICU, intensive care unit; MA, Medicare Advantage; TM, traditional Medicare.

^a^ The other category indicates Alaska Native; American Indian; Asian; Hispanic, unknown race; multiracial; or Pacific Islander. These were grouped due to small sample sizes to protect patient confidentiality.

^b^ *T* test comparing the mean observation months.

^c^ Log-rank test comparing the distribution of survival censored months between TM and MA samples.

^d^ Extracted from the Social Determinants of Health Database maintained by the Agency for Healthcare Research and Quality based on Colorado zip codes. Percentage of the population aged 65 years and older, Gini Index of income inequality (values range from 0 [perfect equality] to 1 [perfect inequality]), and per capita income and education are at the zip code tabulation area level. Per capita income is inflation adjusted to data file year and reported in US dollars. Miles to the nearest medical surgical ICU calculated using population weighted zip centroids.

^e^ The percentage of MA plans in each county was calculated by dividing the number of MA plans in a county by the total number of MA plans in Colorado using data from the MA State/County Penetration files.^38^

Among patients diagnosed with local, regional, or distant NSCLC, those enrolled in MA were older than patients enrolled in TM (Table 1).^38^ Patients enrolled in TM lived in areas with a greater proportion of people 65 years and older and a lower MA penetration rate with a similar number of MA plans available. The number of observation months was similar across the samples. As with CRC, patients enrolled in MA were less likely to be enrolled in Medicaid, and patients enrolled in MA were less likely to be White.^39^

Table 2 reports the unadjusted distribution of patients who received a cancer-directed drug and, conditional on receiving the drug, the distribution of those who received a high-cost drug. Patients enrolled in MA with local or regional CRC are 9.5 percentage points less likely to receive a cancer-directed drug. Conditional on receiving a drug, they were 11.3 percentage points less likely to receive a high-cost drug. For distant CRC, patients enrolled in MA were also less likely to receive a high-cost drug. Patients enrolled in MA were less likely to receive a cancer-directed drug for distant NSCLC and if they received a cancer-directed drug, it was less likely to exceed the $8000 threshold (8.4 percentage points and 8.9 percentage points, respectively).

**Table 2.** Unadjusted Comparisons of Colorectal and Non–Small Cell Lung Cancer Drugs Filled After Diagnosis by Stage and Medicare Plan

Variable	Patients, No. (%)					
	Local/regional			Distant		
	TM	MA	*P* value	TM	MA	*P* value
**Patients with colorectal cancer (n = 1991)**						
Cancer drug(s)						
No	323 (59.6)	764 (69.1)	<.001	35 (25.9)	66 (31.6)	.26
Yes	219 (40.4)	341 (30.9)		100 (74.1)	143 (68.4)	
High-cost threshold						
<$6500	62 (28.3)	135 (39.6)	.01	<10^a^	28 (19.6)	.003
≥$6500	157 (71.7)	206 (60.4)		94 (94.0)	115 (80.4)	
**Patients with non–small cell lung cancer (n = 2249)**						
Cancer drug(s)						
No	295 (63.9)	563 (63.3)	.85	69 (22.5)	183 (30.9)	.01
Yes	167 (36.1)	326 (36.7)		237 (77.5)	409 (69.1)	
High-cost threshold						
<$8000	90 (53.9)	181 (55.5)	.73	64 (27.0)	147 (35.9)	.02
≥$8000	77 (46.1)	145 (44.5)		173 (73.0)	262 (64.1)	

Abbreviations: MA, Medicare Advantage; TM, traditional Medicare.

^a^ Less than 10 is used in this table cell to protect patient confidentiality and adhere to the data use agreement.

Table 3 reports marginal effects from the inverse probability weighted regression estimations for receiving a cancer-directed drug and conditional on receiving a cancer-directed drug, whether the drug exceeded the threshold, adjusted for the covariates in Table 1.^38^ Consistent with the descriptive findings, patients enrolled in MA with local or regional CRC were 6.0 percentage points less likely to receive a cancer-directed drug and 10.0 percentage points less likely to receive a drug that exceeds the threshold. Patients enrolled in MA with distant CRC were 9.0 percentage points less likely to receive a drug that exceeds the threshold.

**Table 3.** Adjusted Marginal Effects of Colorectal Cancer (CRC) and Non–Small Cell Lung Cancer (NSCLC) Drugs by Stage and Medicare Plan

Variable^a^	Local/regional CRC (n = 1647)		Distant CRC (n = 344)		Local/regional NSCLC (n = 1351)		Distant NSCLC (n = 898)	
	ME (95% CI)	*P* value	ME (95% CI)	*P* value	ME (95% CI)	*P* value	ME (95% CI)	*P* value
Cancer drug receipt								
MA	−0.06 (−0.11 to −0.02)	.01	−0.06 (−0.15 to 0.03)	.16	0 (−0.05 to 0.05)	.96	−0.10 (−0.16 to −0.04)	.001
Exceeds high-cost threshold	≥$6500				≥$8000			
No.	560	NA	243	NA	493		646	NA
MA	−0.10 (−0.18 to −0.03)	.01	−0.09 (−0.16 to −0.02)	.01	−0.04 (−0.14 to 0.06)	.43	−0.06 (−0.14 to 0.01)	.10

Abbreviations: MA, Medicare Advantage; ME, marginal effect; NA, not applicable.

^a^ All covariates in Table 1 are included in the model (all footnotes apply).

In the adjusted analysis, patients enrolled in MA with distant NSCLC were 10.0 percentage points less likely to receive cancer-directed drugs. Other differences were not statistically significant, although the coefficient for patients enrolled in MA with distant NSCLC approached significance. Sensitivity analyses restricting the follow-up time to 12 months and using different definitions of high-cost drugs showed similar findings (eTables 4 and 5 in Supplement 1).

## Discussion

In this cohort study, we compared cancer-directed drugs between patients enrolled in TM and MA diagnosed with CRC or NSCLC. We distinguished between local/regional or distant stage disease because treatment options vary and may be forgone altogether when cancer has spread. The evidence suggests that in some cases, patients insured by MA, relative to those insured by TM, are less likely to receive cancer-directed drugs and when they receive these drugs, the drugs are less likely to exceed the threshold for high cost.

MA plans have several ways to reduce utilization of high-cost drugs. For example, MA plans use drug utilization management tools, preauthorization, step treatment protocols, and capitated payments to incentivize clinicians to lower costs.^40,41,42,43,44^ However, a statistically significant difference between patients enrolled in MA and TM in the likelihood of receiving a high-cost drug for distant stage NSCLC is not observed, but the coefficient trends in a negative direction. A possible explanation is that the lower-cost drugs for patients with CRC are recommended for first-line therapy until evidence of disease progression, whereas there are no similar or interchangeable drugs for the high-cost agents to treat patients with NSCLC.

### Limitations

First, we did not examine other treatment modalities including radiation therapy, which may be especially relevant in NSCLC treatment. Second, although we used a rich set of covariates in our analysis and selected only patients residing in urban areas where practice patterns may be similar, we cannot definitively rule out patient selection into TM or MA as an underlying explanation for the patterns we observed. Third, we did not have complete TNM staging on all patients. Therefore, the sample may contain patients for whom surgery without further treatment may be the preferred option. To address this, we removed those with local summary stage as a sensitivity analysis. The results were unchanged. Fourth, there may be concerns about the quality and completeness of MA encounter data^45^ relative to TM claims. Our assessment of the number of treatments lent confidence that encounter and claims data are of similar quality. Fifth, our data were specific to patients residing in urban areas of Colorado and may not generalize to other states, rural areas, or areas with a different MA market. Last, we do not have data on patient preferences that may be correlated with selection into MA or TM, nor do we examine the impact of drug choices on survival.

## Conclusions

Cancer-directed drugs are among the most expensive in the US.^46^ Use of these drugs has led to extreme financial burden among patients.^47,48^ In response to the high cost of health care and medications, different payment models have been sought to control prescribing costs. The results of this cohort study suggest that MA may reduce the use of high-cost drugs when there is an interchangeable alternative to more expensive options. This finding suggests MA may control treatment costs, even if only modestly. When costs are astronomical, as they frequently are with cancer, small savings can be important and substantially reduce costs. Ultimately, the best way to make a large reduction in cancer drug expenditures may be through other measures such as price controls.

## Notes

Supplement 1. eTable 1. Monthly costs and information for systemic anticancer therapies used to treat colorectal and non–small cell lung cancers eTable 2. Codes for systemic anticancer therapies used to treat colorectal and non–small cell lung cancers eTable 3. Systemic anticancer therapies used to treat colorectal and non–small cell lung cancer, stratified by local/regional and distant disease, CCCR-APCD, 2012-2017 eTable 4. Unadjusted comparison of Traditional Medicare and Medicare Advantage colorectal and non–small cell lung cancer cancer-directed drugs filled within 12 months after diagnosis by SEER summary stage, CCCR-APCD, 2012-2021 eFigure 1. Balance plots of the standardized mean differences on each covariate comparing before and after weighting for CRC and NSCLC by SEER summary stage, CCCR-APCD, 2012-2021 eFigure 2. Balance plots of the standardized mean differences on each covariate comparing before and after weighting for CRC and NSCLC who had cancer-directed drugs filled by SEER Summary Stage, CCCR-APCD, 2012-2021 eTable 5. Adjusted marginal effects of cancer-directed drugs filled within 12 months after diagnosis for colorectal and non–small cell lung cancer by SEER summary stage comparing Traditional Medicare and Medicare Advantage using Inverse Probability Weighted Regression, CCCR-APCD, 2012 to 2021 eTable 6. Adjusted marginal effects of cancer-directed drugs filled any time after diagnosis for colorectal and non–small cell lung cancer by SEER summary stage regional and distant comparing Traditional Medicare and Medicare Advantage using Inverse Probability Weighted Regression, CCCR-APCD, 2012 to 2021 eTable 7. Adjusted marginal effect of receiving a high-cost drug after diagnosis among colorectal and non–small cell lung cancer patients who had cancer-directed drugs using Inverse Probability Weighted Regression, CCCR-APCD, 2012 to 2021. Threshold $9,500 (CRC), $12,000 (NSCLC) eTable 8. Adjusted marginal effect of receiving a high-cost drug within 12 months of diagnosis among colorectal and non–small cell lung cancer patients who had cancer-directed drugs using Inverse Probability Weighted Regression, CCCR-APCD, 2012 to 2021. Threshold $9,500 (CRC), $12,000 (NSCLC). eTable 9. Adjusted marginal effects of cancer-directed drugs filled any time after diagnosis for colorectal and non–small cell lung cancer by SEER summary stage (excluded local stage I) comparing Traditional Medicare and Medicare Advantage using Inverse Probability Weighted Regression, CCCR-APCD, 2012 to 2021 eReferences

Supplement 2. Data Sharing Statement
