# Supplementary material for: High-Cost Cancer Drug Use in Medicare Advantage and Traditional Medicare
Source: JAMA Health Forum. 2025 Jan 10;6(1):e244868. doi: 10.1001/jamahealthforum.2024.4868 (PMC11724345; doi:10.1001/jamahealthforum.2024.4868)
Supplement: Supplement 1. — eTable 1. Monthly costs and information for systemic anticancer therapies used to treat colorectal and non–small cell lung cancers eTable 2. Codes for systemic anticancer therapies used to treat colorectal and non–small cell lung cancers eTable 3. Systemic anticancer therapies used to treat colorectal and non–small cell lung cancer, stratified by local/regional and distant disease, CCCR-APCD, 2012-2017 eTable 4. Unadjusted comparison of Traditional Medicare and Medicare Advantage colorectal and non–small cell lung cancer cancer-directed drugs filled within 12 months after diagnosis by SEER summary stage, CCCR-APCD, 2012-2021 eFigure 1. Balance plots of the standardized mean differences on each covariate comparing before and after weighting for CRC and NSCLC by SEER summary stage, CCCR-APCD, 2012-2021 eFigure 2. Balance plots of the standardized mean differences on each covariate comparing before and after weighting for CRC and NSCLC who had cancer-directed drugs filled by SEER Summary Stage, CCCR-APCD, 2012-2021 eTable 5. Adjusted marginal effects of cancer-directed drugs filled within 12 months after diagnosis for colorectal and non–small cell lung cancer by SEER summary stage comparing Traditional Medicare and Medicare Advantage using Inverse Probability Weighted Regression, CCCR-APCD, 2012 to 2021 eTable 6. Adjusted marginal effects of cancer-directed drugs filled any time after diagnosis for colorectal and non–small cell lung cancer by SEER summary stage regional and distant comparing Traditional Medicare and Medicare Advantage using Inverse Probability Weighted Regression, CCCR-APCD, 2012 to 2021 eTable 7. Adjusted marginal effect of receiving a high-cost drug after diagnosis among colorectal and non–small cell lung cancer patients who had cancer-directed drugs using Inverse Probability Weighted Regression, CCCR-APCD, 2012 to 2021. Threshold $9,500 (CRC), $12,000 (NSCLC) eTable 8. Adjusted marginal effect of receiving a high-cost drug within 12 months of diagno [file jamahealthforum-e244868-s001.pdf]

Supplemental Online Content

Bradley CJ, Liang R, Lindrooth RC, Sabik LM, Perraillon MC. High-cost cancer drug use in Medicare Advantage and Traditional Medicare. *JAMA Health Forum.* 2025;6(1):e244868. doi:10.1001/jamahealthforum.2024.4868

eTable 1. Monthly costs and information for systemic anticancer therapies used to treat colorectal and non-small cell lung cancers

eTable 2. Codes for systemic anticancer therapies used to treat colorectal and non-small cell lung cancers

eTable 3. Systemic anticancer therapies used to treat colorectal and non-small cell lung cancer, stratified by local/regional and distant disease, CCCR-APCD, 2012-2017

eTable 4. Unadjusted comparison of Traditional Medicare and Medicare Advantage colorectal and non-small cell lung cancer cancer-directed drugs filled within 12 months after diagnosis by SEER summary stage, CCCR-APCD, 2012-2021

eFigure 1. Balance plots of the standardized mean differences on each covariate comparing before and after weighting for CRC and NSCLC by SEER summary stage, CCCR-APCD, 2012-2021

eFigure 2. Balance plots of the standardized mean differences on each covariate comparing before and after weighting for CRC and NSCLC who had cancer-directed drugs filled by SEER Summary Stage, CCCR-APCD, 2012-2021

eTable 5. Adjusted marginal effects of cancer-directed drugs filled within 12 months after diagnosis for colorectal and non-small cell lung cancer by SEER summary stage comparing Traditional Medicare and Medicare Advantage using Inverse Probability Weighted Regression, CCCR-APCD, 2012 to 2021

eTable 6. Adjusted marginal effects of cancer-directed drugs filled any time after diagnosis for colorectal and non-small cell lung cancer by SEER summary stage regional and distant comparing Traditional Medicare and Medicare Advantage using Inverse Probability Weighted Regression, CCCR-APCD, 2012 to 2021

eTable 7. Adjusted marginal effect of receiving a high-cost drug after diagnosis among colorectal and non-small cell lung cancer patients who had cancer-directed drugs using Inverse Probability Weighted Regression, CCCR-APCD, 2012 to 2021. Threshold $9,500 (CRC), $12,000 (NSCLC)

© 2025 Bradley CJ et al. *JAMA Health Forum*.

eTable 8. Adjusted marginal effect of receiving a high-cost drug within 12 months of diagnosis among colorectal and non-small cell lung cancer patients who had cancer-directed drugs using Inverse Probability Weighted Regression, CCCR-APCD, 2012 to 2021. Threshold $9,500 (CRC), $12,000 (NSCLC).

eTable 9. Adjusted marginal effects of cancer-directed drugs filled any time after diagnosis for colorectal and non-small cell lung cancer by SEER summary stage (excluded local stage I) comparing Traditional Medicare and Medicare Advantage using Inverse Probability Weighted Regression, CCCR-APCD, 2012 to 2021

eReferences

This supplemental material has been provided by the authors to give readers additional information about their work.

© 2025 Bradley CJ et al. *JAMA Health Forum*.

eTable 1. Monthly costs and information of systemic anticancer therapies used to treat colorectal and non-small cell lung cancers

Colorectal Cancer	Medi -care Part[1]	Year of FDA Approval	Monthly Cost (adjusted, 2020)	Above threshold of $6,500	Above threshold of $9,500	Purpose
Adagrasib	D	2022	$17,678	Yes	Yes	Target cells with KRAS gene changes
Bevacizumab	B	2004	$6,069	No	No	Target blood vessel formation (VEGF)
Capecitabine	D	1998	$1,659	No	No	Chemotherapy
Cetuximab	B	2004	$12,969	Yes	Yes	Target cancer cells with EGFR changes
Dostarlimab	D	2021	$14,302	Yes	Yes	Immunotherapy PD-1 inhibitors
Encorafenib[2]	D	2020	$8,050	Yes	No	Target cells with BRAF gene changes
Entrectinib	D	2019	$17,191	Yes	Yes	Target cells with NTRK gene changes
Fam-trastuzumab deruxtecan	B	2019	$13,367	Yes	Yes	Target cells with HER2 changes
Fluorouracil Injection[2]	B	1970	$62	No	No	Chemotherapy
Fruquintinib	D	2023	$23,224	Yes	Yes	Target blood vessel formation (VEGF)
Ipilimumab	B	2011	$43,672	Yes	Yes	Immunotherapy CTLA-4 inhibitor
Irinotecan Hydrochloride	B	1996	$8,788	Yes	No	Chemotherapy
Lapatinib	D	2007	$3,659	No	No	Target cells with HER2 changes
Larotrectinib	D	2018	$34,172	Yes	Yes	Target cells with NTRK gene changes
Leucovorin Calcium[2]	B	1987	$567	No	No	Chemotherapy
Nivolumab	B	2014	$13,594	Yes	Yes	Immunotherapy PD-1 inhibitors
Oxaliplatin	B	2002	$8,505	Yes	No	Chemotherapy
Panitumumab	B	2006	$10,259	Yes	Yes	Target cancer cells with EGFR changes
Pembrolizumab	B	2014	$10,101	Yes	Yes	Immunotherapy PD-1 inhibitors
Pertuzumab	B	2012	$8,745	Yes	No	Target cells with HER2 changes
Ramucirumab	B	2014	$14,314	Yes	Yes	Target blood vessel formation (VEGF)
Regorafenib	D	2012	$11,415	Yes	Yes	Other targeted therapy drugs
Selpercatinib	D	2020	$20,823	Yes	Yes	Target cells with RET gene changes
Sotorasib	D	2021	$17,282	Yes	Yes	Target cells with KRAS gene changes
Transtuzumab	B	1998	$5,093	No	No	Target cells with HER2 changes
Trifluridine and Tipiracil Hydrochloride	D	2015	$12,839	Yes	Yes	Chemotherapy

© 2025 Bradley CJ et al. *JAMA Health Forum*.

Tucatinib	D	2020	$18,700	Yes	Yes	Target cells with HER2 changes
Ziv-Aflibercept	B	2012	$12,470	Yes	Yes	Target blood vessel formation (VEGF)

**NSCLC**	**Medi care Part**	**Year of FDA Approval**	**Monthly Cost (adjusted, 2020)**	**Above threshold of $6,000**	**Above threshold of $12,000**	**Purpose**
Adagrasib	D	2022	$17,678	Yes	Yes	Target cells with KRAS gene changes
Ado-trastuzumab emtansine	B	2013	$11,815	Yes	No	Target cells with HER2 gene changes
Afatinib	D	2013	$6,233	Yes	No	Target cells with EGFR gene changes
Alectinib	D	2015	$13,607	Yes	Yes	Target cells with ALK gene changes
Amivantamab-vmjw	B	2021	$24,714	Yes	Yes	Target cells with an exon 20 mutation (EGFR inhibitors)
Atezolizumab	B	2016	$14,163	Yes	Yes	Immunotherapy - PD-1/PD-L1 inhibitors
Bevacizumab	B	2004	$6,069	Yes	No	Target tumor blood vessel growth (angiogenesis VEGF inhibitors)
Binimetinib	D	2018	$15,514	Yes	Yes	Target cells with BRAF gene changes
Brigatinib	D	2017	$14,575	Yes	Yes	Target cells with ALK gene changes
Capmatinib Hydrochloride	D	2020	$19,440	Yes	Yes	Target cells with MET gene changes
Carboplatin	B	1991	$1,634	No	No	Chemotherapy (available as generic)
Cemiplimab-rwlc	B	2018	$14,298	Yes	Yes	Immunotherapy - PD-1/PD-L1 inhibitors
Ceritinib	D	2014	$14,916	Yes	Yes	Target cells with ALK gene changes/target cells with ROS1 gene changes
Cisplatin	B	1978	$496	No	No	Chemotherapy (available as generic)
Crizotinib	D	2011	$11,133	Yes	No	Target cells with ALK gene changes/target cells with ROS1 gene changes/target cells with MET gene changes
Dabrafenib	D	2013	$8,535	Yes	No	Target cells with BRAF gene changes
Dacomitinib	D	2018	$12,919	Yes	Yes	Target cells with EGFR gene changes
Docetaxel	B	1996	$4,307	No	No	Chemotherapy
Doxorubicin Hydrochloride	B	1987	$1,187	No	No	Chemotherapy
Durvalumab	B	2017	$11,811	Yes	No	Immunotherapy - PD-1/PD-L1 inhibitors
Encorafenib[2]	D	2023	$8,050	Yes	No	Target cells with BRAF gene changes

© 2025 Bradley CJ et al. *JAMA Health Forum*.

Entrectinib	D	2019	$17,191	Yes	Yes	Target cells with ROS1 gene changes/target cells with NTRK gene changes
Erlotinib Hydrochloride	D	2004	$5,719	No	No	Target cells with EGFR gene changes
Everolimus	D	2009	$7,061	Yes	No	Target cells with HER2 gene changes
Etoposide	B	1996	$1,130	No	No	Chemotherapy (available as generic)
Fam-Trastuzumab Deruxtecan-nxki	B	2019	$13,367	Yes	Yes	Target cells with HER2 gene changes
Gefitinib	D	2003	$2,262	No	No	Target cells with EGFR gene changes
Gemcitabine Hydrochloride	B	1996	$3,513	No	No	Chemotherapy
Ipilimumab	B	2011	$43,672	Yes	Yes	Immunotherapy - CTLA-4 inhibitors
Lorlatinib	D	2018	$16,728	Yes	Yes	Target cells with ALK gene changes/target cells with ROS1 gene changes/target cells with NTRK gene changes
Methotrexate Sodium	D	2001/2017	$59.86/$855.11	No	No	Chemotherapy
Mobocertinib Succinate	D	2021	$24,137	Yes	Yes	Target cells with an exon 20 mutation
Necitumumab[2]	B	2015	$14,046	Yes	Yes	Monoclonal antibody used for squamous cell NSCLC
Nivolumab	B	2014	$13,594	Yes	Yes	Immunotherapy - PD-1/PD-L1 inhibitors
Osimertinib Mesylate	D	2015	$14,073	Yes	Yes	Target cells with EGFR gene changes/target cells with the T790M mutation
Paclitaxel	B	1994	$4,564	No	No	Chemotherapy
Paclitaxel Albumin-stabilized Nanoparticle Formulation	B	2005	$7,474	Yes	No	Chemotherapy
Pembrolizumab	B	2014	$10,101	Yes	No	Immunotherapy - PD-1/PD-L1 inhibitors
Pemetrexed Disodium	B	2004	$6,969	Yes	No	Chemotherapy
Pralsetinib	D	2020	$19,452	Yes	Yes	Target cells with RET gene changes
Ramucirumab	B	2014	$14,314	Yes	Yes	Target tumor blood vessel growth (angiogenesis VEGF inhibitors)
Repotrectinib	D	2023	$22,865	Yes	Yes	Target cells with ROS1 gene changes
Selpercatinib	D	2020	$20,823	Yes	Yes	Target cells with RET gene changes
Sotorasib	D	2021	$17,282	Yes	Yes	Target cells with KRAS gene changes
Tepotinib Hydrochloride	D	2021	$20,177	Yes	Yes	Target cells with MET gene changes
Trametinib Dimethyl Sulfoxide	D	2013	$9,770	Yes	No	Target cells with BRAF gene changes

© 2025 Bradley CJ et al. *JAMA Health Forum*.

Tremelimumab-actl	B	2022	$37,402	Yes	Yes	Immunotherapy - CTLA-4 inhibitors
Vinorelbine Tartrate	B	1994	$1,807	No	No	Chemotherapy

Notes: Sources for data are as of March 15, 2024: Systemic anticancer therapies approved for colorectal cancer extracted from Colorectal Cancer Treatment (PDQ®)–Patient Version on National Cancer Institute website (https://www.cancer.gov/about-cancer/treatment/drugs/colorectal) and Treating Colorectal Cancer from American Cancer Society (https://www.cancer.org/cancer/types/colon-rectal-cancer/treating.html). Systemic anticancer therapies approved for non-small cell lung cancer extracted from National Cancer Institute website (https://www.cancer.gov/about-cancer/treatment/drugs/lung#2) and Treating Non-Small Cell Lung Cancer from American Cancer Society (https://www.cancer.org/cancer/types/lung-cancer/treating-non-small-cell.html).

Monthly costs were adjusted in 2020 dollars and extracted from https://www.drugpricinglab.org/issue/launch-price-tracker/.

[1] "Part" refers to type or setting of chemotherapy covered by Medicare. Medicare Part B covers some oral chemotherapy medications, as well as intravenous (IV) chemotherapy given in a doctor's office or clinic. Medicare Part D covers some chemotherapy medications that Part B does not, as well as other anticancer drugs.

[2] The monthly cost adjusted 2020 for fluorouracil injection (part B drug approved in 1970), leucovorin calcium (part B drug approved in 1987), and encorafenib (part D drug approved in 2018) were not available in the Drug Pricing Lab. We estimated the monthly prices using the price calculation methods from Drug Pricing Lab. The relevant cost is applied to a 12-week dosing regimen for an "average" adult weighing 70kg, or with a body surface area of 1.7 meters squared and divided by 2.77 to arrive at a monthly price (there are, on average, 2.77 months in 12 weeks). For Part B drugs, we can apply the 2020 Average Sale Price (ASP) from CMS as basis cost. We followed the Mayo Regimen Standard Dose of fluorouracil injection and leucovorin calcium. For fluorouracil injection, the average ASP for J9190 in 2020 was $1.596 per 500mg. The Mayo Regimen is 425 mg/m^2 per day for five days every 4 or 5 weeks. For leucovorin calcium, the average ASP for J0640 in 2020 was $3.088 per 50mg. The standard dose is 200 mg/m^2 per day for five days over 4 or 5 weeks. For encorafenib, FDA approves Braftovi (encorafenib) in combination with cetuximab for the treatment of BRAFV600E-mutant metastatic colorectal cancer (CRC) after prior therapy on April 8, 2020. And then FDA Approves Braftovi (encorafenib) with Mektovi (binimetinib) for Metastatic NSCLC with a BRAF V600E Mutation on October 11, 2023. We used the monthly cost of 300 mg once daily calculated in the paper "Evaluation of the Cost-effectiveness of Doublet Therapy in Metastatic BRAF Variant Colorectal Cancer"[1] because it is a Part D drug and there is no ASP from CMS for it. For necitumumab, the average ASP for J9295 in 2020 was 5.735 per mg. The standard dose is 800mg (absolute dose) as an intravenous infusion over 60 minutes on Days 1 and 8 of each 3-week cycle. Drug necitumumab approved in 2015 and Medicare has reimbursed at 106% of the average sales price (ASP) for Part B drugs since 2005, so we adjusted the price with 106% of the ASP.

© 2025 Bradley CJ et al. *JAMA Health Forum*.

eTable 2. Codes for systemic anticancer therapies used to treat colorectal and non-small cell lung cancers

Systemic anticancer therapy	HCPCS Code	NDC Code
Adagrasib		80739-0812-12 80739-0812-18
Ado-trastuzumab emtansine	C9131 J9354	50242-0087-01 50242-0088-01
Afatinib		00597-0137-30 00597-0137-90 00597-0138-30 00597-0138-95 00597-0141-30
Alectinib		50242-0130-01 50242-0130-86
Amivantamab-vmjw	C9083 J9061	57894-0501-01
Atezolizumab	C9483 J9022	50242-0918-01 50242-0918-86 50242-0917-01 50242-0917-86
Bevacizumab	C9257 C9214 J9035 Q2024 Q5118 S0116	00069-0315-01 00069-0342-01 50242-0060-01 50242-0060-10 50242-0061-01 50242-0061-10 55513-0206-01 55513-0207-01 70121-1754-01 70121-1754-07 70121-1755-01 70121-1755-07 72606-0011-01 72606-0011-10 72606-0012-01 72606-0012-10
Bevacizumab	C9257 C9214 J9035 Q2024 Q5118 S0116	00069-0315-01 00069-0342-01 50242-0060-01 50242-0060-10 50242-0061-01 50242-0061-10 55513-0206-01 55513-0207-01
Binimetinib		70255-0010-02 70255-0010-03
Brigatinib		76189-0113-18 76189-0113-21
Capecitabine	J8520 J8521	00054-0271-21 00054-0272-23 00093-7473-06 00093-7474-89 16714-0467-01 16714-0468-01 64980-0276-06 64980-0277-12 65162-0843-06 65162-0844-06 65162-0844-16 65162-0844-50 59651-0205-08 59651-0205-10 59651-0204-10 59651-0204-60 00004-1100-20 00004-1100-75 00004-1101-50 00004-1101-51 00004-1101-75 69539-0019-60 69539-0019-99 69539-0020-92 69539-0020-99 72205-0007-92 72485-0204-60 72485-0205-12 68001-0487-06 54868-5260-00 54868-5260-01 54868-5260-02 54868-5260-03 54868-5260-05 54868-5260-09 55111-0496-05 55111-0496-60 55111-0497-04 55111-0497-05 59923-0721-60 59923-0722-12 62756-0238-18 62756-0238-86 62756-0238-88 62756-0239-18 62756-0239-20 62756-0239-83 68001-0488-07 70756-0816-22 72606-0554-01 70756-0815-60 72606-0555-01 72205-0006-60 16729-0072-12 16729-0073-29 60687-0149-94 00179-0149-70 00179-0195-70 42291-0166-60 42291-0167-12 42291-0190-60 42291-0191-12 50268-0154-13 53808-0411-01 54868-4143-00 63759-3000-01 63759-3001-01 69097-0948-08 69097-0949-03 00378-2511-91 00378-2512-78 51079-0510-05
Capmatinib Hydrochloride		00078-0709-56 00078-0709-94 00078-0716-56 00078-0716-94
Carboplatin	J9045 (CPT code can be Null)	00703-3249-11 47335-0150-40 47335-0151-40 68083-0190-01 68083-0191-01 68083-0192-01 68083-0193-01 71288-0100-05 71288-0100-15 71288-0100-45 71288-0100-51 00015-3210-30 00015-3211-30 00015-3212-30 00015-3213-30 00015-3214-30 00015-3215-30 00015-3216-30 61703-0360-18 61703-0360-22 61703-0360-50 00703-4239-01 00703-4239-81 00703-4244-01 00703-4244-81 00703-4246-01 00703-4246-81 57277-0105-05 57277-0106-15 67457-0491-54 67457-0492-15 67457-0493-46 67457-0494-61 67457-0608-20 55150-0386-01

© 2025 Bradley CJ et al. *JAMA Health Forum*.

		16729-0295-12 16729-0295-31 16729-0295-33 16729-0295-34 00703-4248-01 00703-4248-81 00703-4248-91 61703-0339-18 61703-0339-22 61703-0339-50 61703-0339-56 25021-0202-05 25021-0202-15 25021-0202-45 25021-0202-51 47335-0284-40 47781-0603-20 47781-0604-27 47781-0605-94 47781-0606-94 50742-0445-05 50742-0446-15 57277-0107-45 66758-0047-01 66758-0047-02 66758-0047-03 66758-0047-04 50742-0447-45 63323-0172-05 63323-0172-15 63323-0172-45 63323-0172-60 69448-0005-12 69448-0005-31 69448-0005-33 69448-0005-34 69448-0005-38 50742-0448-60
Cemiplimab-rwlc	J9113	61755-0008-01
Ceritinib		00078-0694-48 00078-0694-84 00078-0640-70
Cetuximab	C9215 J9055	66733-0948-23 66733-0958-23
Cisplatin	C9418 J9060 J9062	00703-5747-11 00703-5748-11 16729-0288-11 16729-0288-38 44567-0509-01 44567-0510-01 44567-0511-01 63323-0103-51 63323-0103-64 63323-0103-65 68001-0283-27 68001-0283-32 68083-0162-01 68083-0163-01 44567-0530-01 00015-3070-97 00015-3072-20 00015-3072-97 67457-0424-10 67457-0425-51 70860-0206-50 70860-0206-51 00069-0081-01 00069-0084-07 47781-0609-25 47781-0610-23 61126-0003-10 61126-0004-01 61126-0004-02
Crizotinib		00069-8140-20 00069-8141-20
Dabrafenib		00078-0681-66 00078-0682-66 00173-0846-08 00173-0847-08 00173-0847-61 00173-0847-65
Dacomitinib		00069-0197-30 00069-2299-30 00069-1198-30 63539-0197-90
Docetaxel	J9170 J9171	00075-8005-01 00409-0366-01 00409-0367-01 00955-1020-01 00955-1021-04 00955-1022-08 25021-0222-01 25021-0222-04 25021-0222-07 43598-0258-11 43598-0610-40 43598-0611-11 45963-0765-52 66758-0050-01 66758-0050-02 66758-0050-03 66758-0950-02 66758-0950-03 66758-0950-04 25021-0245-01 25021-0245-04 50742-0431-08 50742-0463-16 70121-1221-01 70121-1222-01 70121-1223-01 43066-0001-01 43066-0006-01 43066-0010-01 00069-9141-11 00069-9141-22 00069-9142-11 00069-9142-22 00075-8001-20 00075-8001-80 00069-9144-11 00069-9144-22 00409-0369-01 67457-0531-02 67457-0532-08 00143-9204-01 00143-9205-01 43598-0389-57 47335-0323-40 47335-0895-40 47335-0939-40 72485-0216-08 72485-0215-04 72485-0214-01 71288-0143-02 71288-0144-08 71288-0144-16 71288-0150-95 71288-0151-95 71288-0151-96 00409-7870-01 00409-0365-01 00409-1732-01 00409-4235-01 00409-5068-01 55150-0378-01 55150-0379-01 55150-0380-01 68083-0401-01 68083-0400-01 68083-0399-01 00409-0201-02 00409-0201-10 00409-0201-20 00409-0201-25 00409-0201-26 00409-0201-27 00409-0368-01 16729-0231-63 16729-0231-64 16729-0231-65 16729-0267-63 16729-0267-64 16729-0267-65 43598-0259-40 45963-0734-52 45963-0734-54 45963-0734-74 47335-0285-41 67457-0533-16 67457-0781-08 69097-0372-47 70700-0174-22 70700-0175-22 70700-0176-22 00075-8003-01 00075-8004-04 00703-5720-01 00703-5730-01 16714-0465-01

© 2025 Bradley CJ et al. *JAMA Health Forum*.

		16714-0500-01 16729-0120-49 16729-0228-50 39822-2120-01 39822-2180-01 39822-2200-01 42367-0121-21 42367-0121-25 42367-0121-29 45963-0781-74 45963-0790-56 50742-0428-02 57884-3021-00 63739-0932-11 63739-0971-17 69097-0369-32 69097-0371-37 00409-0016-01 00409-2026-01
Dostarlimab	C9082 J9272	00173-0898-03
Doxorubicin Hydrochloride	C9415 J9000 J9001 J9002 Q2048 Q2049 Q2050	00069-3031-20 00069-3032-20 00069-3033-20 00069-4004-05 00069-4015-10 00069-4026-25 00069-4037-01 00143-9546-01 00143-9547-01 00143-9548-01 00143-9548-10 00143-9549-01 00143-9549-10 00409-0124-01 16714-0742-01 16714-0856-01 43598-0283-35 43598-0541-25 45963-0733-55 45963-0733-57 45963-0733-60 45963-0733-68 47335-0049-40 47335-0050-40 63323-0101-61 63323-0883-05 63323-0883-10 63323-0883-30 68083-0248-01 68083-0249-01 68083-0250-01 70121-1219-01 00143-9275-01 00143-9277-01 43598-0682-35 43598-0683-25 68001-0345-36 68001-0345-26 55390-0237-01 55390-0238-01 00013-1116-83 00013-1136-91 00013-1146-91 00013-1156-79 00013-1176-87 00013-1266-83 00013-1286-83 00069-0170-01 00069-0171-01 00069-3030-20 00069-3034-20 67457-0394-00 67457-0394-10 00338-0067-01 00338-0063-01 16714-0001-01 49315-0008-03 49315-0009-07 00143-9092-01 00143-9093-01 72603-0103-01 72603-0200-01 70710-1530-01 70710-1531-01 68001-0492-36 68001-0493-26 67457-0393-00 67457-0393-54 67457-0395-25 67457-0396-10 67457-0436-50 67457-0478-10 00069-4030-01 00069-4031-01 00069-4032-01 00069-4033-01 00069-4034-01 00703-5040-01 00703-5043-01 00703-5043-03 00703-5046-01 25021-0207-05 25021-0207-25 25021-0207-51 00574-0931-25 00574-0930-10 47335-0082-50 47335-0083-50 47781-0256-17 47781-0256-18 47781-0256-19 53150-0314-01 53150-0314-10 53150-0315-01 53150-0317-01 53150-0320-01 53150-0320-10 59676-0960-01 59676-0960-02 59676-0966-01 59676-0966-02 62756-0826-40 62756-0827-40
Durvalumab	C9492 J9173 (J9999)	00310-4500-12 00310-4611-50
Encorafenib		70255-0020-01 70255-0025-01 70255-0025-02 70255-0025-03 70255-0025-03 70255-0025-05 70255-0025-06
Encorafenib (used w/ cetuximab)		70255-0020-01 70255-0025-01 70255-0025-03 70255-0025-05 70255-0025-06
Entrectinib	C9399 J8999	50242-0091-30 50242-0091-86 50242-0092-01 50242-0092-86 50242-0094-47 50242-0094-90
Erlotinib Hydrochloride		50242-0062-01 50242-0063-01 50242-0064-01 54868-5290-00 54868-5447-00 42292-0051-05 42292-0052-05 42292-0053-05 51991-0890-33 51991-0891-33 51991-0892-33 69539-0091-30 69539-0092-30 69539-0090-30 72205-0081-30 72205-0080-30 72205-0082-30 72485-0218-30 72485-0219-30 72485-0217-30 46708-0565-30 46708-0565-90 46708-0566-30 46708-0566-90 46708-0567-30 46708-0567-90 62332-0565-30 62332-0565-90 62332-0566-30 62332-0566-90 62332-0567-30 62332-0567-90 00378-7131-93 00378-7132-93 00378-7133-93 54868-5474-00 69189-0063-01

© 2025 Bradley CJ et al. *JAMA Health Forum*.

Etoposide	C9414 C9425 J8560 J9181 J9182	00378-3266-94 00703-5653-01 55390-0291-01 16729-0114-08 16729-0114-11 16729-0114-31 16729-0114-32 68001-0265-22 68001-0265-23 68001-0265-24 68001-0265-25 68001-0265-26 68001-0265-27 00015-3404-20 16729-0262-31 16729-0262-32 63323-0104-05 63323-0104-25 63323-0104-50 63323-0104-06 55390-0292-01 55390-0293-01 55390-0491-01 55390-0492-01 55390-0493-01 00703-5656-01 00703-5656-91 00703-5657-01 00703-5657-91
Everolimus	J7527 J8561	00093-7766-24 00093-7767-24 00093-7768-24 00054-0480-13 00054-0480-14 00054-0481-13 00054-0481-14 00054-0497-13 00054-0497-14 00378-3096-85 00378-3097-85 00378-3098-85 49884-0119-91 49884-0125-91 49884-0127-91 51991-0821-28 51991-0822-28 51991-0823-28 51991-0379-60 00378-0005-85 00378-0006-85 00378-0007-85 51991-0824-28 70377-0010-22 70377-0011-22 70377-0012-22 70377-0013-22 63850-0058-01 63850-0059-01 63850-0060-01 63850-0061-01 00054-0482-13 00078-0566-51 00078-0566-61 00078-0567-51 00078-0567-61 00078-0594-51 00078-0594-61 00078-0620-51 00078-0620-61 00078-0626-51 00078-0626-61 00078-0627-51 00078-0627-61 00078-0628-51 00078-0628-61
Fam-trastuzumab deruxtecan	J9358	65597-0406-01
Fam-Trastuzumab Deruxtecan-nxki	J9358	65597-0406-01
Fluorouracil Injection	J9190	00069-0169-01 00069-0169-02 00069-0173-01 00069-0173-02 00069-0174-01 00069-0176-01 00187-3204-47 00378-4791-06 00703-3015-13 00703-3018-12 00703-3019-12 10139-0063-01 10139-0063-10 10139-0063-11 10139-0063-12 10139-0063-20 10139-0063-50 16729-0276-03 16729-0276-05 16729-0276-11 16729-0276-38 16729-0276-67 16729-0276-68 16729-0542-35 21695-0829-40 25021-0215-98 25021-0215-99 43547-0258-01 46708-0751-50 50742-0423-10 50742-0481-20 50742-0482-50 50742-0483-99 51672-4063-01 51672-4118-02 51672-4118-05 51672-4118-06 51862-0362-40 52549-4118-02 52549-4118-05 52549-4118-06 54868-0951-00 54868-6293-00 62332-0751-50 63323-0117-10 63323-0117-18 63323-0117-19 63323-0117-20 63323-0117-28 63323-0117-51 63323-0117-58 63323-0117-59 63323-0117-61 63323-0117-68 63323-0117-69 66530-0249-40 66758-0044-01 66758-0044-03 66758-0054-01 66758-0054-02 68001-0266-27 68001-0266-30 68001-0266-31 68001-0266-32 68001-0524-30 68001-0524-31 68001-0525-27 68001-0525-32 68071-2961-04 68083-0292-01 68083-0293-01 68682-0004-31 68682-0085-31 70700-0186-23 70700-0187-23 70700-0188-22 70700-0189-22
Fruquintinib		63020-0210-21 63020-0225-21
Gefitinib	J8565	00310-0482-30 00310-0482-93
Gemcitabine Hydrochloride	J9198 J9201	00002-7502-01 00781-3282-75 00781-3283-79 16729-0092-03 16729-0117-11 16729-0118-38 55390-0391-10 55390-0391-50 68083-0148-01 68083-0149-01 16714-0909-01 16714-0930-01 71288-0113-10 71288-0114-50 25021-0209-50 25021-0234-10 25021-0235-50 42236-0001-01 42236-0002-01 70860-0204-10

© 2025 Bradley CJ et al. *JAMA Health Forum*.

		70860-0205-50 71288-0117-06 71288-0117-28 71288-0117-54 72485-0221-02 72485-0222-10 72485-0223-20 00002-7501-01 23155-0213-31 23155-0528-31 25021-0208-10 45963-0619-59 67457-0616-10 67457-0617-30 67457-0618-10 00069-3857-10 00069-3858-10 00069-3859-10 00591-3562-79 00591-3563-55 23155-0214-31 23155-0483-31 23155-0484-31 23155-0529-31 45963-0612-57 45963-0620-60 47335-0153-40 47335-0154-40 60505-6113-06 60505-6114-00 60505-6115-02 67457-0462-01 67457-0463-02 67457-0464-20 68001-0282-22 68001-0282-25 68001-0282-26 68001-0282-27 69097-0313-37 69097-0314-42
Ipilimumab	C9284 J9228	00003-2327-11 00003-2328-22
Ipilimumab	C9284 J9228	00003-2327-11 00003-2328-22
Irinotecan Hydrochloride	C9474 J9206	00009-1111-01 00009-1111-02 00009-7529-03 00009-7529-04 00009-7529-05 00143-9583-01 00143-9701-01 00143-9702-01 00703-4432-11 00703-4432-81 00703-4434-11 00703-4434-81 15054-0043-01 16714-0027-01 16714-0131-01 16714-0725-01 16714-0726-01 23155-0179-31 23155-0179-32 25021-0214-02 25021-0214-05 25021-0230-02 25021-0230-05 45963-0614-51 45963-0614-55 45963-0614-56 45963-0614-81 45963-0614-85 47335-0937-40 47335-0953-40 50742-0401-02 50742-0402-05 55150-0352-01 55150-0353-01 55150-0354-01 55150-0355-01 59923-0702-02 59923-0702-05 59923-0714-02 59923-0715-05 59923-0716-15 60505-6272-01 61703-0349-09 61703-0349-16 61703-0349-36 63323-0193-02 63323-0193-05 63323-0193-52 63323-0193-55 66758-0048-01 66758-0048-02 68001-0284-25 68001-0284-34 68001-0425-35 68001-0426-22 68001-0480-22 68001-0480-35 68083-0381-01 68083-0382-01 69171-0398-01 70700-0169-22 70700-0170-22 72485-0211-02 72485-0212-05 72485-0213-15
Lapatinib		00078-0671-19 00173-0752-00 68180-0801-36
Larotrectinib		50419-0390-01 50419-0391-01 50419-0392-01 50419-0393-03 71777-0390-01 71777-0391-01 71777-0392-01
Leucovorin Calcium/Levoleucovorin/Levoleucovorin Calcium	J0640 J0641 J0642	00054-4496-13 00054-4496-25 00054-4497-05 00054-4497-10 00054-4498-10 00054-4499-11 00054-8496-19 00143-9552-01 00143-9553-01 00143-9554-01 00143-9555-01 00143-9558-01 00555-0484-01 00555-0484-02 00555-0484-05 00555-0484-18 00555-0485-04 00555-0485-27 00591-4130-54 00703-5140-01 00703-5145-01 00703-5145-91 00781-3201-94 14335-0340-01 14335-0341-01 16714-0890-01 16714-0915-01 25021-0813-10 25021-0813-66 25021-0814-30 25021-0814-67 25021-0815-30 25021-0815-67 25021-0816-30 25021-0816-67 25021-0828-50 42806-0133-21 42806-0133-24 42806-0134-24 42806-0134-34 42806-0358-01 42806-0358-30 42806-0359-25 43598-0771-11 43598-0773-11 45963-0762-57 50742-0181-01 50742-0181-30 50742-0182-12 50742-0182-24 50742-0183-24 50742-0184-25 50742-0464-50 50742-0494-17 50742-0495-25 51079-0581-01 51079-0581-06 51079-0582-01 51079-0582-05 52125-0018-02 52125-0453-02 53270-0101-01 54868-3310-00 54868-3310-01 54868-3310-02 54868-3310-03 54868-3310-04 54868-5915-00 55390-0009-01 55390-0051-10

© 2025 Bradley CJ et al. *JAMA Health Forum*.

		55390-0052-10 55390-0053-01 55390-0054-01 55390-0818-10 55390-0824-01 55390-0825-01 55390-0826-01 60687-0227-11 60687-0227-94 63323-0631-10 63323-0631-50 63323-0710-50 63323-0710-59 63323-0711-00 67457-0528-10 67457-0529-20 67457-0530-35 67457-0600-20 67457-0601-30 68001-0285-22 68001-0285-28 68001-0285-29 68001-0285-36 68001-0285-37 68001-0285-38 68001-0285-39 68001-0285-40 68001-0286-38 68001-0286-39 68001-0416-36 68001-0417-37 68001-0418-38 68083-0278-01 68083-0279-01 68152-0101-00 68152-0102-01 68152-0102-02 68152-0112-01 68152-0114-01 69315-0184-01 69315-0184-03 69315-0185-12 69315-0185-24 69315-0186-24 69315-0187-25 70121-1099-01 70121-1572-01 70436-0116-82 70436-0117-80 70436-0118-80 70436-0120-80 70436-0209-80 70436-0210-80 71205-0908-00 71205-0908-11 71205-0908-30 71205-0908-55 71205-0908-60 71205-0908-90 71288-0104-10 71288-0105-18 71288-0105-25 71288-0160-10 71288-0161-20 71288-0162-30 71288-0163-30 71288-0164-50 72266-0120-01 72266-0121-01 72893-0004-01 72893-0006-01
Lorlatinib		00069-0227-01 00069-0231-01
Methotrexate Sodium	J8610 J9250 J9260	00054-4550-15 00054-4550-25 00054-8550-25 10139-0062-02 10139-0062-10 10139-0062-40 49349-0314-02 49349-0406-02 54868-3826-00 54868-3826-01 54868-3826-02 54868-3826-03 54868-3826-04 54868-3826-05 54868-3826-06 54868-3826-07 54868-3826-08 54868-3826-09 54868-4716-00 55390-0031-10 55390-0032-10 55390-0033-10 55390-0034-10 55390-0143-01 61703-0351-59 61703-0352-07 61786-0417-02 61786-0417-03 61786-0417-07 00703-3673-01 54569-1818-09 63323-0122-50 63323-0122-59 63323-0123-02 63323-0123-10 67457-0465-00 67457-0465-08 00069-0204-01 00069-0204-10 21695-0111-00 21695-0111-30 63629-1472-01 63629-1472-02 63629-1472-03 63629-1472-04 63629-1472-05 63629-1472-06 63629-1472-07 63629-1472-08 50090-2345-04 50090-2345-09 63323-0121-02 66758-0040-01 66758-0040-02 66758-0040-07 66758-0040-08 66758-0041-01 70518-1251-00 70518-2711-00 75840-0111-00 75840-0111-01
Mobocertinib Succinate		63020-0040-12 63020-0040-90
Necitumumab	C9475 J9295	00002-7716-01
Nivolumab	J9299 C9453 (C9399/J9999/J8999)	00003-3772-11 00003-3774-12 00003-3734-13 00003-3756-14
Nivolumab	C9453 J9299 (C9399 J8999 J9999)	00003-3772-11 00003-3774-12 00003-3734-13 00003-3756-14
Osimertinib Mesylate		00310-1350-30 00310-1350-95 00310-1349-30
Oxaliplatin	C9205 J9263	00703-3985-01 00703-3986-01 00781-3315-70 00781-3317-80 00781-9315-70 00781-9317-80 00955-1725-10 00955-1727-20 00955-1731-10 00955-1733-20 16714-0727-01 16714-0728-01 25021-0233-10 25021-0233-20 45963-0611-53 45963-0611-59 57277-0001-05 57277-0002-10 63323-0175-30 63323-0176-50

© 2025 Bradley CJ et al. *JAMA Health Forum*.

		63323-0211-10 63323-0212-20 67184-0508-01 67184-0509-01 67184-0510-01 68083-0170-01 68083-0171-01 71288-0101-10 71288-0101-20 16729-0332-03 16729-0332-05 63323-0650-10 63323-0650-17 63323-0650-20 63323-0650-27 00069-0070-01 00069-0074-01 68083-0176-01 68083-0177-01 60505-6132-06 60505-6132-07 60505-6132-08 67184-0501-01 67184-0502-01 68001-0468-36 68001-0468-37 72266-0125-01 72266-0125-10 72266-0126-01 72266-0126-10 72266-0162-01 72266-0161-01 72603-0101-01 72603-0301-01 79672-0825-02 79672-0826-02 79672-0018-01 79672-0019-01 00024-0591-20 00024-0590-10 00069-0067-01 25021-0211-20 25021-0212-50 61703-0361-35 61703-0362-50 61703-0363-18 61703-0363-22 63323-0750-10 63323-0750-17 63323-0750-20 63323-0750-27 67457-0442-20 67457-0469-10 68001-0341-36 68001-0341-37 69097-0274-37 69097-0353-78 00069-1010-01 12516-0592-04 43066-0014-01 43066-0018-01 45963-0637-49 45963-0638-58 47335-0046-40 47335-0047-40 47335-0176-40 47335-0178-40 47781-0591-22 47781-0592-29 50742-0405-10 50742-0406-20 51991-0218-98 51991-0922-98 51991-0923-98 67457-0468-50 67457-0476-10 70860-0201-10 70860-0201-20
Paclitaxel	C9127 C9431 J9265 J9267	55390-0114-05 55390-0304-05 55390-0314-05 68083-0178-01 68083-0179-01 68083-0180-01 70860-0200-05 70860-0200-17 70860-0200-50 47781-0595-07 00703-3216-01 00703-3216-81 00703-3217-01 00703-3213-01 00703-3213-81 00703-3218-01 00703-3218-81 16714-0137-01 69539-0158-01 69539-0159-01 69539-0157-01 72205-0063-01 72205-0062-01 72205-0061-01 00703-4764-01 00703-4764-81 00703-4768-01 00703-4768-81 44567-0504-01 44567-0505-01 44567-0506-01 45963-0613-53 45963-0613-56 45963-0613-59 45963-0613-83 45963-0613-86 45963-0613-89 61703-0342-09 61703-0342-22 61703-0342-50 63323-0763-05 63323-0763-06 63323-0763-16 63323-0763-17 63323-0763-50 63323-0763-52 00069-0076-01 00069-0078-01 00069-0079-01 00703-4766-01 00703-4766-81 00703-4767-01 25021-0213-05 25021-0213-17 25021-0213-50 68001-0516-27 47781-0593-07 47781-0594-07 51991-0937-98 51991-0938-98 70860-0215-66 70860-0215-67 70860-0215-68 66758-0043-01 66758-0043-02 66758-0043-03 67457-0434-51 67457-0449-17 67457-0471-52
Paclitaxel Albumin-stabilized Nanoparticle Formulation	J9264	68817-0134-50
Panitumumab	C9235 J9303	55513-0954-01 55513-0956-01 55513-0955-01 59703-0956-01
Pembrolizumab	C9027 J9271	00006-3026-02 00006-3026-04 00006-3029-02
Pembrolizumab	C9027 J9271	00006-3026-02 00006-3026-04 00006-3029-02
Pemetrexed Disodium	C9213 J9304 J9305	00002-7640-01 00002-7623-01 00002-7623-61 67184-0503-01
Pertuzumab	C9292 J9306	50242-0145-01
Pralsetinib		72064-0210-12 72064-0210-60 72064-0210-90 50242-0210-12 50242-0210-60 50242-0210-83 50242-0210-86 50242-0210-90
Ramucirumab	C9025 J9308	00002-7669-01 00002-7678-01

© 2025 Bradley CJ et al. *JAMA Health Forum*.

Regorafenib		50419-0171-00 50419-0171-01 50419-0171-03 50419-0171-04 50419-0171-06
Repotrectinib		00003-4040-12 00003-4040-60
Selpercatinib		00002-2980-26 00002-2980-60 00002-3977-60
Sotorasib		55513-0488-02 55513-0488-24
Tepotinib Hydrochloride		44087-5000-03 44087-5000-06
Trametinib Dimethyl Sulfoxide		00078-0666-15 00173-0848-13 00173-0848-65 00078-0668-15 00173-0849-13 00173-0858-13
Transtuzumab (w/ tucatinib)	J9355 Q5112 Q5113 Q5114 Q5116 Q5117	00006-5033-02 00069-0305-01 00069-0308-01 50242-0132-01 50242-0132-10 50242-0134-68 50242-0333-01 55513-0132-01 55513-0141-01 55513-0164-01 63459-0303-43 63459-0305-47 67457-0847-44 67457-0991-15 78206-0147-01 83257-0001-11 83257-0003-01 83257-0004-12
Tremelimumab-actl	C9147 J9347	00310-4505-25 00310-4535-30
Trifluridine and Tipiracil Hydrochloride		64842-1020-01 64842-1020-02 64842-1020-03 64842-1025-01 64842-1025-02 64842-1025-03
Tucatinib		51144-0001-60 51144-0002-12 51144-0002-60
Vinorelbine Tartrate	C9440 J9390	55390-0069-01 55390-0070-01 00008-0045-01 00008-0045-02 00069-0099-01 00069-0103-03 00069-0205-10 00069-0205-50 00703-4182-01 00703-4182-91 00703-4183-01 00703-4183-91 25021-0204-01 25021-0204-05 45963-0607-55 45963-0607-56 66758-0045-01 66758-0045-02 67457-0431-11 67457-0479-53 67457-0481-01 67457-0482-05 50742-0420-01 50742-0427-05 61703-0341-06 61703-0341-09 64370-0532-01 64370-0532-02
Ziv-Aflibercept	C9296 J9400	00024-5841-01 00024-5840-01 00024-5840-03

Note: HCPCS, Healthcare Common Procedure Drug Coding, NDC, National Drug Code. Source for data is from Cancer Medications Enquiry Database (CanMED).[2]

© 2025 Bradley CJ et al. *JAMA Health Forum*.

eTable 3. Systemic anticancer therapies used to treat colorectal and non-small cell lung cancer, stratified by local/regional and distant disease, CCCR-APCD, 2012-2017

Systemic anticancer therapy	Year approved	Monthly cost adjusted 2020	Frequency, local/regional[a]			Frequency, distant[a]		
			All	TM	MA	All	TM	MA
**Colorectal**								
Fluorouracil Injection[b]	1970	$62	3097	1275	1822	2893	1502	1391
Leucovorin Calcium[b]	1987	$567	2888	1199	1689	2737	1458	1279
Oxaliplatin	2002	$8,505	2426	1097	1329	1524	753	771
Bevacizumab	2004 (1st line) 2006 (2nd line)	$6,069	1477	572	905	2121	1097	1024
Capecitabine	1998 (Part D)	$1659	1453	518	935	606	202	404
Irinotecan Hydrochloride	1996	$8,788	518	203	315	1020	535	485
Cetuximab	2004	$12,969	131	78	53	299	81	218
Pembrolizumab	2020 (for CRC)	$10,101	112	62	50	94	37	57
Trastuzumab	1998	$5093	>105	<10	105	<10	<10	-
Trifluridine and Tipiracil Hydrochloride	2015 (Part D)	$12,839	75	41	34	71	22	49
Nivolumab	2017 (for CRC)	$13,594	70	70	-	<10	-	<10
Panitumumab	2006	$10,259	>34	<10	34	175	59	116
Regorafenib	2012 (Part D)	$11,415	>15	<10	15	>15	<10	15
Pertuzumab	2012	$8,745	<10	<10	<10	-	-	-
Ziv-Aflibercept	2012	$12,470	<10	<10	-	48	18	30
Ramucirumab	2015 (for CRC)	$14,314	<10	<10	-	<10	-	<10
Fam-Trastuzumab Deruxtecan-nxki	2019	$13,367	<10	-	<10	-	-	-
Ipilimumab	2018 (combined with Nivolumab)	$43,672	-	-	-	<10	<10	-
Encorafenib[c]	2020 (Part D for CRC)	$8,050	-	-	-	<10	<10	<10
Lapatinib	2007 (Part D)	$3,659	-	-	-	<10	<10	-
**Non-small cell Lung Cancer**								
Carboplatin	1991	$1,634	1617	541	1076	1713	624	1089

© 2025 Bradley CJ et al. *JAMA Health Forum*.

Paclitaxel	1994	$4,564	1306	397	909	755	283	472
Durvalumab	2017	$11,811	1159	442	717	115	16	99
Pembrolizumab	2014	$10,101	839	271	568	2745	1123	1622
Pemetrexed Disodium	2004 (2nd line) 2008 (1st line)	$6,969	575	211	364	2237	881	1356
Gemcitabine Hydrochloride	1996	$3,513	311	86	225	686	356	330
Cisplatin	1978	$496	295	117	178	64	28	36
Etoposide	1983/1996 (1st line for SCLC)	$470 (1983) $1130 (1996)	261	99	162	330	110	220
Nivolumab	2014	$13,594	244	60	184	822	152	670
Bevacizumab	2006 (for NSCLC)	$6,069	217	74	143	406	158	248
Osimertinib Mesylate	2015 (Part D)	$14,073	188	75	113	415	186	229
Vinorelbine Tartrate	1994	$1,807	161	33	128	>24	<10	24
Docetaxel	1996	$4,307	107	40	67	312	100	212
Erlotinib Hydrochloride	2004 (Part D)	$5,719	>75	<10	75	388	145	243
Paclitaxel Albumin-stabilized Nanoparticle Formulation	2005	$7,474	79	19	60	95	18	77
Methotrexate Sodium	2001/2017 (Part D)	$60 (2001) $855 (2017)	51	25	26	>10	<10	10
Alectinib	2015 (2nd line) 2017(1st line ALK-positive) (Part D)	$13,607	48	-	48	423	252	171
Crizotinib	2011 (Part D)	$11,133	45	-	45	136	34	102
Atezolizumab	2016	$14,163	30	15	15	103	60	43
Ramucirumab	2014	$14,314	>21	21	<10	45	19	26
Afatinib	2013 (Part D)	$6,233	-	-	-	>88	88	<10
Dacomitinib	2018 (Part D)	$12,919	10	10	-	<10	<10	-
Capmatinib Hydrochloride	2020 (Part D)	$19,440	<10	-	<10	<10	<10	-
Ado-trastuzumab emtansine	2013	$11,815	<10	-	<10	<10	<10	-
Doxorubicin Hydrochloride	1987	$1,187	<10	<10	<10	<10	<10	<10
Lorlatinib	2018 (Part D)	$16,728	<10	-	<10	11	<10	<10
Ipilimumab	2011	$43,672	<10	-	<10	>12	<10	12
Gefitinib	2003	$2262	<10	<10	-	-	-	-
Fam-Trastuzumab Deruxtecan-nxki	2019	$13,367	<10	-	<10	>18	<10	18

© 2025 Bradley CJ et al. *JAMA Health Forum*.

Amivantamab-vmjw	2021	$24,714	<10	-	<10	<10	<10	-
Everolimus	2009 (Part D)	$7,061	<10	-	<10	-	-	-
Selpercatinib	2020 (Part D)	$20,823	-	-	-	23	-	23
Entrectinib	2019 (Part D)	$17,191	-	-	-	10	-	10
Ceritinib	2014 (Part D)	$14,916	-	-	-	<10	-	<10
Sotorasib	2021 (Part D)	$17,282	-	-	-	<10	<10	-
Necitumumab[b]	2015	$14,046	-	-	-	<10	-	<10
Mobocertinib Succinate	2021 (Part D)	$24,137	-	-	-	<10	<10	-
Dabrafenib	2013 (Part D)	$8,535	-	-	-	<10	<10	-
Trametinib Dimethyl Sulfoxide	2017 (for NSCLC, Part D)	$9,770	-	-	-	<10	<10	-

Notes: TM, Traditional Medicare, MA, Medicare Advantage, CRC, colorectal cancer, NSCLC, non-small cell lung cancer, CCCR-APCD=Colorado Central Cancer Registry-All Payer Claims Data. Frequency was defined as number of claims, which are distinct by the date of service and corresponding codes. For colorectal cancer, there were 560 (34% of 1647) local/regional patients and 243 (71% of 344) distant stage patients who received any colorectal-specific systematic anticancer therapies after diagnosis. For non-small cell lung cancer, there were 493 (37% of 1351) local/regional patients and 646 (72% of 898) distant stage patients who ever received any NSCLC-specific systematic anticancer therapies after diagnosis. Sources for data are:

Pemetrexed NSCLC second line: http://theoncologist.alphamedpress.org/content/10/6/363.long; Pemetrexed NSCLC first line: http://theoncologist.alphamedpress.org/content/14/9/930.long; Bevacizumab NSCLC: http://theoncologist.alphamedpress.org/content/12/6/713.full; Bevacizumab CRC first line: chrome-extension://efaidnbmnnnibpcajpcglclefindmkaj/https://www.gene.com/download/pdf/avastin_prescribing.pdf; Bevacizumab CRC second line: https://theoncologist.onlinelibrary.wiley.com/doi/10.1634/theoncologist.12-3-356; Nivolumab NSCLC: https://theoncologist.onlinelibrary.wiley.com/doi/full/10.1634/theoncologist.2015-0507; Nivolumab CRC: https://www.cancer.gov/news-events/cancer-currents-blog/2017/nivolumab-fda-colorectal; Alectinib: https://www.cancer.gov/news-events/cancer-currents-blog/2017/alectinib-fda-untreated-lung-cancer; Trametinib Dimethyl Sulfoxide: https://theoncologist.onlinelibrary.wiley.com/doi/10.1634/theoncologist.2017-0642; Ipilimumab CRC: https://theoncologist.onlinelibrary.wiley.com/doi/10.1634/theoncologist.2019-0129; Pembrolizumab CRC: https://aacrjournals.org/clincancerres/article/27/17/4680/671637/FDA-Approval-Summary-Pembrolizumab-for-the-First; Ramucirumab CRC: https://theoncologist.onlinelibrary.wiley.com/doi/10.1634/theoncologist.2015-0028.

Monthly costs were adjusted in 2020 dollars and data was extracted from https://www.drugpricinglab.org/issue/launch-price-tracker/. Frequency reports reflect number of patients in the study sample who received the drugs.

[a] Local/regional and distant stages were defined using the SEER Summary Stage from 2000 and 2018.

[b] The monthly cost adjusted 2020 for fluorouracil injection (part B drug approved in 1970), leucovorin calcium (part B drug approved in 1987), necitumumab (part B drug approved in 2015), and encorafenib (part D drug approved in 2018) were not available in the Drug Pricing Lab. We estimated the monthly prices using the price calculation methods from Drug Pricing Lab and using the 2020 Average Sale Price (ASP) from CMS for Part B drugs as basis cost. The relevant cost is applied to a 12-week dosing regimen for an "average" adult weighing 70kg, or with a body surface area of 1.7 meters squared and divided by 2.77 to arrive at a monthly price (there are, on average, 2.77 months in 12 weeks). We followed the Mayo Regimen Standard Dose of fluorouracil injection and leucovorin calcium. For fluorouracil injection, the average ASP for J9190 in 2020 was $1.596 per 500mg. The Mayo Regimen is 425 mg/m^2 per day for five days every 4 or 5 weeks. For leucovorin calcium, the average ASP for J0640 in 2020 was $3.088 per 50mg. The standard dose is 200 mg/m^2 per day for five days over 4 or 5 weeks.

[c] For encorafenib, we used the monthly cost of 300 mg once daily calculated in the paper "Evaluation of the Cost-effectiveness of Doublet Therapy in Metastatic BRAF Variant Colorectal Cancer". For necitumumab, the average ASP for J9295 in 2020 was 5.735 per mg. The standard dose is 800mg (absolute dose) as an intravenous infusion over 60 minutes on Days 1 and 8 of each 3-week cycle. Drug necitumumab approved in 2015 and Medicare has reimbursed at 106% of the average sales price (ASP) for Part B drugs since 2005, so we adjusted the price with 106% of the ASP.

© 2025 Bradley CJ et al. *JAMA Health Forum*.

eTable 4. Unadjusted comparison of Traditional Medicare and Medicare Advantage colorectal and non-small cell lung cancer cancer-directed drugs filled within 12 months after diagnosis by SEER summary stage, CCCR-APCD, 2012-2021

	**Local/Regional**			**Distant**		
	TM	MA	P value	TM	MA	P value
			**Colorectal Cancer (N=1991)**			
No drug	351 (64.8)	800 (72.4)	0.002	36 (26.7)	71 (34.0)	0.153
CRC drug(s)	191 (35.2)	305 (27.6)		99 (73.3)	138 (66.0)	
**Threshold**						
<$6500	56 (29.3)	125 (41.0)	0.009	8 (8.1)	31 (22.5)	0.003
>=$6500	135 (70.7)	180 (59.0)		91 (91.9)	107 (77.5)	
			**Non-small cell Lung Cancer (N=2249)**			
No drug	318 (68.8)	618 (69.5)	0.796	73 (23.9)	192 (32.4)	0.008
NSCLC drug(s)	144 (31.2)	271 (30.5)		233 (76.1)	400 (67.6)	
**Threshold**						
<$8000	94 (65.3)	182 (67.2)	0.699	75 (32.2)	169 (42.3)	0.012
>=$8000	50 (34.7)	89 (32.8)		158 (67.8)	231 (57.7)	

Notes**:** SEER, Surveillance, Epidemiology, End-Results, TM, traditional Medicare; MA, Medicare Advantage; CCCR, Colorado Central Cancer Registry; APCD, All Payer Claims Database. Percent of sample size shown in parentheses.

© 2025 Bradley CJ et al. *JAMA Health Forum*.

eFigure 1. Balance plots of the standardized mean differences on each covariate comparing before and after weighting for CRC and NSCLC by SEER summary stage, CCCR-APCD, 2012-2021

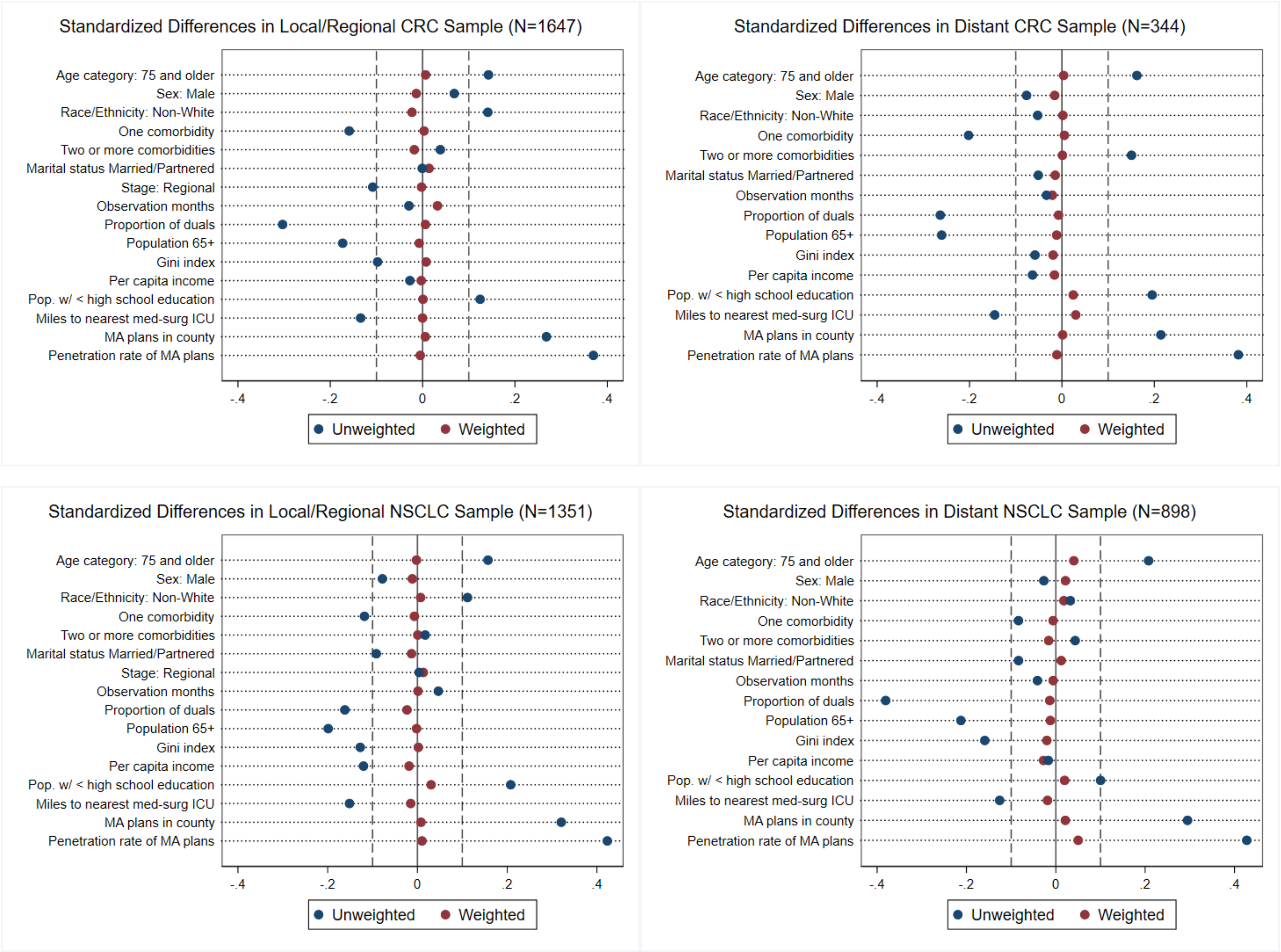

© 2025 Bradley CJ et al. *JAMA Health Forum*.

eFigure 2. Balance plots of the standardized mean differences on each covariate comparing before and after weighting for CRC and NSCLC who had cancer-directed drugs filled by SEER Summary Stage, CCCR-APCD, 2012-2021

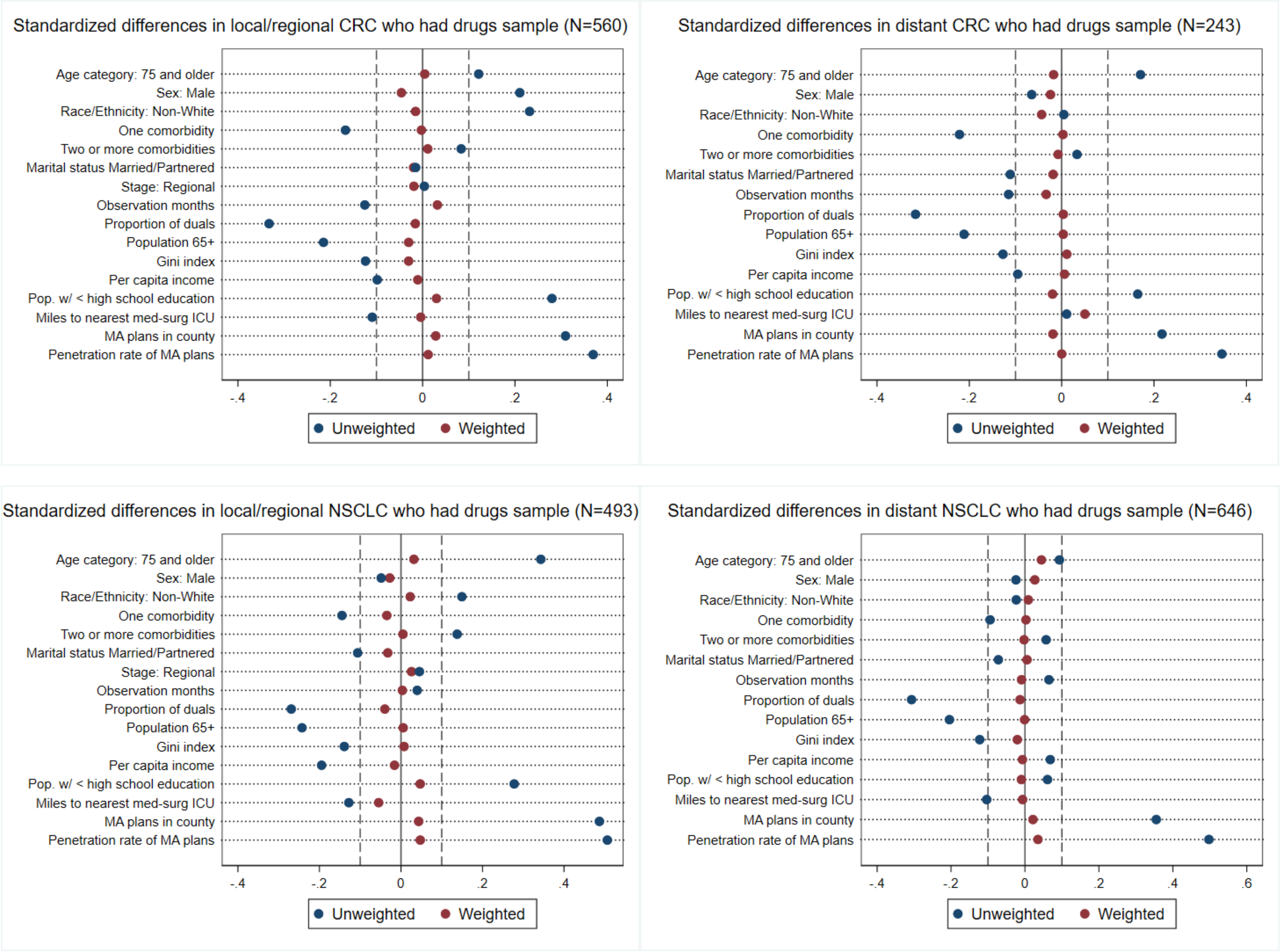

© 2025 Bradley CJ et al. *JAMA Health Forum*.

eTable 5. Adjusted marginal effects of cancer-directed drugs filled within 12 months after diagnosis for colorectal and non-small cell lung cancer by SEER summary stage comparing Traditional Medicare and Medicare Advantage using Inverse Probability Weighted Regression, CCCR-APCD, 2012 to 2021

**Variable[a]**	**Local/Regional CRC (N=1,647)**		**Distant CRC (N=344)**		**Local/Regional NSCLC (N=1351)**		**Distant NSCLC (N=898)**	
	**ME (95% CI)**	***P* value**	**ME (95% CI)**	***P* value**	**ME (95% CI)**	***P* value**	**ME (95% CI)**	***P* value**
**Insurance**								
TM	Reference		Reference		Reference		Reference	
MA	-0.05 [-0.09, 0]	0.032	-0.08 [-0.17, 0.01]	0.076	-0.02 [-0.06, 0.03]	0.508	-0.14 [-0.21, -0.06]	<0.001
**Exceeds threshold**		≥$6500				≥$8,000		
	**(N=496)**		**(N=237)**		**(N=415)**		**(N=633)**	
TM	Reference		Reference		Reference		Reference	
MA	-0.10 [-0.18, -0.02]	0.013	-0.10 [-0.18, -0.02]	0.011	0.02 [-0.08, 0.12]	0.711	-0.07 [-0.15, 0.01]	0.079

Notes**:** SEER, Surveillance, Epidemiology, End Results, ME, marginal effect; CRC, colorectal cancer; NSCLC, non-small cell lung cancer. TM, traditional Medicare; MA, Medicare Advantage; CCCR, Colorado Central Cancer Registry; APCD, All Payer Claims Database. [a]All covariates in Table 1 are included in the model. Footnotes to Table 1 apply.

© 2025 Bradley CJ et al. *JAMA Health Forum*.

eTable 6. Adjusted marginal effects of cancer-directed drugs filled any time after diagnosis for colorectal and non-small cell lung cancer by SEER summary stage regional and distant comparing Traditional Medicare and Medicare Advantage using Inverse Probability Weighted Regression, CCCR-APCD, 2012 to 2021

**Variable**[1]	**Regional CRC (N=849)**		**Distant CRC (N=344)**		**Regional NSCLC (N=566)**		**Distant NSCLC (N=898)**	
	**ME (95% CI)**	***P* value**	**ME (95% CI)**	***P* value**	**ME (95% CI)**	***P* value**	**ME (95% CI)**	***P* value**
**Insurance**								
TM	Reference		Reference		Reference		Reference	
MA	-0.07 [-0.14, 0]	0.043	-0.06 [-0.15, 0.03]	0.163	0.01 [-0.07, 0.09]	0.857	-0.10 [-0.16, -0.04]	0.001
**Exceeds threshold**		≥$6500				≥$8,000		
	**(N=448)**		**(N=243)**		**(N=355)**		**(N=646)**	
TM	Reference		Reference		Reference		Reference	
MA	-0.09 [-0.17, -0.01]	0.034	-0.09 [-0.16, -0.02]	0.008	-0.04 [-0.15, 0.07]	0.428	-0.06 [-0.14, 0.01]	0.098

Notes**:** SEER, Surveillance, Epidemiology, End Results, ME, marginal effect; CRC, colorectal cancer; NSCLC, non-small cell lung cancer. TM, traditional Medicare; MA, Medicare Advantage; CCCR, Colorado Central Cancer Registry; APCD, All Payer Claims Database. All covariates in Table 1 are included in the model. Footnotes to Table 1 apply.

© 2025 Bradley CJ et al. *JAMA Health Forum*.

eTable 7. Adjusted marginal effect of receiving a high-cost drug[a] after diagnosis among colorectal and non-small cell lung cancer patients who had cancer-directed drugs using Inverse Probability Weighted Regression, CCCR-APCD, 2012 to 2021. Threshold $9,500.

Variable[b]	Local/Regional CRC[c] (N=560)		Distant CRC (N=243)		Local/Regional NSCLC (N=493)		Distant NSCLC (N=646)	
	ME (95% CI)	*P* value	ME (95% CI)	*P* value	ME (95% CI)	*P* value	ME (95% CI)	*P* value
**Insurance**	>=$9,500				>=$12,000			
TM	1 [Reference]		1 [Reference]		1 [Reference]		1 [Reference]	
MA	-0.01 [-0.06, 0.04]	0.640	0.05 [-0.07, 0.17]	0.396	-0.02 [-0.09, 0.05]	0.643	-0.02 [-0.09, 0.05]	0.612

Notes**:** SEER, Surveillance, Epidemiology, End Results, ME, marginal effect; CRC, colorectal cancer; NSCLC, non-small cell lung cancer. TM, traditional Medicare; MA, Medicare Advantage; CCCR, Colorado Central Cancer Registry; APCD, All Payer Claims Database. [a] The threshold for high-cost drugs is $9,500 for CRC and $12,000 for NSCLC. [b] All covariates in Table 1 are included in the model. Footnotes to Table 1 apply. [c] The results listed are from the outcome model without adjusting for the proportion of dually eligible participants due to small sample sizes during the study period.

eTable 8. Adjusted marginal effect of receiving a high-cost drug[a] within 12 months of diagnosis among colorectal and non-small cell lung cancer patients who had cancer-directed drugs using Inverse Probability Weighted Regression, CCCR-APCD, 2012 to 2021. Threshold $9,500.

Variable[b]	Local/Regional CRC[c] (N=496)		Distant CRC[d] (N=237)		Local/Regional NSCLC[c] (N=415)		Distant NSCLC (N=633)	
	ME (95% CI)	*P* value	ME (95% CI)	*P* value	ME (95% CI)	*P* value	ME (95% CI)	*P* value
**Insurance**	>=$9,500				>=$12,000			
TM	1 [Reference]		1 [Reference]		1 [Reference]		1 [Reference]	
MA	-0.02 [-0.06, 0.02]	0.282	0 [-0.1, 0.09]	0.93	0.01 [-0.03, 0.06]	0.631	-0.01 [-0.07, 0.05]	0.677

Notes**:** SEER, Surveillance, Epidemiology, End Results, ME, marginal effect; CRC, colorectal cancer; NSCLC, non-small cell lung cancer. TM, traditional Medicare; MA, Medicare Advantage; CCCR, Colorado Central Cancer Registry; APCD, All Payer Claims Database. [a] The threshold for high-cost drugs is $9,500 for CRC and $12,000 for NSCLC. [b] All covariates in Table 1 are included in the model. Footnotes to Table 1 apply. [c] The results listed are from the outcome model without adjusting for the proportion of dually eligible participants and race and ethnicity categories due to small sample sizes during the study period. [d] The results listed are from the outcome model without adjusting for the proportion of dually eligible participants due to small sample sizes during the study period.

© 2025 Bradley CJ et al. *JAMA Health Forum*.

eTable 9. Adjusted marginal effects of cancer-directed drugs filled any time after diagnosis for colorectal and non-small cell lung cancer by SEER summary stage (excluded local stage I) comparing Traditional Medicare and Medicare Advantage using Inverse Probability Weighted Regression, CCCR-APCD, 2012 to 2021

**Variable[1]**	**Regional CRC (N=849)**		**Distant CRC (N=344)**		**Regional NSCLC (N=566)**		**Distant NSCLC (N=898)**	
	**ME (95% CI)**	***P* value**	**ME (95% CI)**	***P* value**	**ME (95% CI)**	***P* value**	**ME (95% CI)**	***P* value**
**Insurance**								
TM	Reference		Reference		Reference		Reference	
MA	-0.07 [-0.14, 0]	0.043	-0.06 [-0.15, 0.03]	0.163	0.01 [-0.07, 0.09]	0.857	-0.10 [-0.16, -0.04]	0.001
**Exceeds threshold**		≥$6500				≥$8,000		
	**(N=448)**		**(N=243)**		**(N=355)**		**(N=646)**	
TM	Reference		Reference		Reference		Reference	
MA	-0.09 [-0.17, -0.01]	0.034	-0.09 [-0.16, -0.02]	0.008	-0.04 [-0.15, 0.07]	0.428	-0.06 [-0.14, 0.01]	0.098

Notes**:** SEER, Surveillance, Epidemiology, End Results, ME, marginal effect; CRC, colorectal cancer; NSCLC, non-small cell lung cancer. TM, traditional Medicare; MA, Medicare Advantage; CCCR, Colorado Central Cancer Registry; APCD, All Payer Claims Database. All covariates in Table 1 are included in the model. Footnotes to Table 1 apply.

© 2025 Bradley CJ et al. *JAMA Health Forum*.

eReferences

1. Patel KK, Stein S, Lacy J, O'Hara M, Huntington SF. Evaluation of the Cost-effectiveness of Doublet Therapy in Metastatic BRAF Variant Colorectal Cancer. *JAMA Netw Open*. Jan 4 2021;4(1):e2033441. doi:10.1001/jamanetworkopen.2020.33441

2. National Cancer Institute Surveillance Epidemiology and End Results Program. Cancer Medications Enquiry Database (CanMED). Surveillance Research Program SEER website tool. Version 1.21.1, 2024. Accessed March 27, 2024. https://seer.cancer.gov/oncologytoolbox/

3. Patel KK, Stein S, Lacy J, O'Hara M, Huntington SF. Evaluation of the Cost-effectiveness of Doublet Therapy in Metastatic BRAF Variant Colorectal Cancer. *JAMA Network Open*. 2021;4(1):e2033441-e2033441. doi:10.1001/jamanetworkopen.2020.33441

© 2025 Bradley CJ et al. *JAMA Health Forum*.
