# Supplementary material for: High-Cost Cancer Drug Use in Medicare Advantage and Traditional Medicare
Source: JAMA Health Forum. 2025 Jan 10;6(1):e244868. doi: 10.1001/jamahealthforum.2024.4868 (PMC11724345; doi:10.1001/jamahealthforum.2024.4868)
Supplement: Supplement 2. — Data Sharing Statement [file jamahealthforum-e244868-s002.pdf]

Data Sharing Statement

Bradley. High-Cost Cancer Drug Use in Medicare Advantage and Traditional Medicare. *JAMA Health Forum*. Published January 10, 2025. doi:10.1001/jamahealthforum.2024.4868

Data
Data available: Yes
Data types: Deidentified participant data
How to access data: Data are available under the conditions of the Data Use Agreement.
When available: With publication

Supporting Documents
Document types: None

Additional Information
Who can access the data: Data may be shared under the conditions of the data use agreement with the Center for Improving Value in Health Care.
Types of analyses: For any purpose.
Mechanisms of data availability: Data may be shared under the conditions of the data use agreement with the Center for Improving Value in Health Care.
